# Interactome analysis reveals endocytosis and membrane recycling of EpCAM during differentiation of embryonic stem cells and carcinoma cells

**DOI:** 10.1016/j.isci.2021.103179

**Published:** 2021-09-27

**Authors:** Min Pan, Vera Kohlbauer, Alexandra Blancke Soares, Henrik Schinke, Yuanchi Huang, Gisela Kranz, Tanja Quadt, Matthias Hachmeister, Olivier Gires

**Affiliations:** 1 Department of Otorhinolaryngology, The First Affiliated Hospital of Chongqing Medical University, Yuzhong District, Chongqing, China; 2 Department of Otorhinolaryngology, Head and Neck Surgery, University Hospital, LMU Munich, Munich, Germany; 3 Clinical Cooperation Group “Personalized Radiotherapy in Head and Neck Cancer”, Helmholtz Zentrum München, Neuherberg, Germany

**Keywords:** Cell biology, Stem cells research, Cancer

## Abstract

Transmembrane epithelial cell adhesion molecule (EpCAM) is expressed in epithelia, carcinoma, teratoma, and embryonic stem cells (ESCs). EpCAM displays spatiotemporal patterning during embryogenesis, tissue morphogenesis, cell differentiation, and epithelial-to-mesenchymal transition (EMT) in carcinomas. Potential interactors of EpCAM were identified in murine F9 teratoma cells using a stable isotope labeling with amino acids in cell culture-based proteomic approach (n = 77, enrichment factor >3, p value ≤ 0.05). Kyoto Encyclopedia of Genes and Genomes and gene ontology terms revealed interactions with regulators of endosomal trafficking and membrane recycling, which were further validated for Rab5, Rab7, and Rab11. Endocytosis and membrane recycling of EpCAM were confirmed in mF9 cells, E14TG2α ESC, and Kyse30 carcinoma cells. Reduction of EpCAM during mesodermal differentiation and TGFβ-induced EMT correlated with enhanced endocytosis and block or reduction of recycling in ESCs and esophageal carcinoma cells. Hence, endocytosis and membrane recycling are means of regulation of EpCAM protein levels during differentiation of ESC and EMT induction in carcinoma cells.

## Highlights

• Proteomic screen identifies various interaction clusters of EpCAM
• EpCAM is subject to retrograde transport and recycling to the plasma membrane
• Endocytosis regulates EpCAM levels in mesodermal differentiation and EMT

## Graphical abstract

**Figure:**
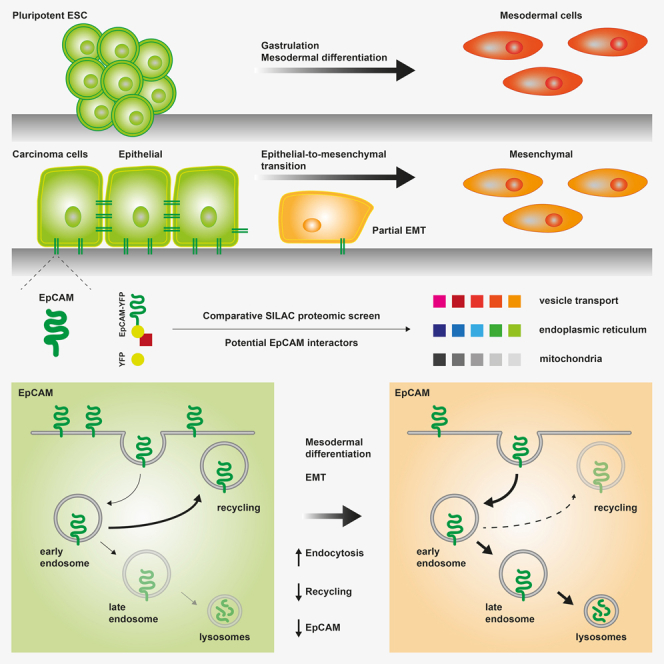

## Introduction

Epithelial cell adhesion molecule (EpCAM) was initially identified as an antigen expressed on colon carcinoma cells that induced a humoral response in mice (Herlyn et al., 1979; Koprowski et al., 1979). EpCAM was later described to be strongly and frequently expressed in the majority of carcinomas (Baeuerle and Gires, 2007; Went et al., 2004), in pluripotent embryonic stem cells (ESCs) (Gonzalez et al., 2009; Lu et al., 2010; Ng et al., 2009), and hepatic progenitors (Dolle et al., 2015; Schmelzer et al., 2006, 2007). EpCAM is composed of an extracellular domain, a single type I transmembrane domain, and a short intracellular domain (Balzar et al., 1999). Functional implications of this transmembrane glycoprotein range from cell adhesion and junction (Ladwein et al., 2005; Litvinov et al., 1994a, 1994b; Wu et al., 2013), migration and morphogenesis (Gaiser et al., 2012; Maghzal et al., 2010, 2013; Slanchev et al., 2009), tissue integrity (Gaston et al., 2021; Guerra et al., 2012; Kozan et al., 2015; Lei et al., 2012; Salomon et al., 2017; Sivagnanam et al., 2008), proliferation (Munz et al., 2004; Osta et al., 2004), and signal transduction (Chaves-Perez et al., 2013; Maetzel et al., 2009) to differentiation and stem cell pluripotency (Gonzalez et al., 2009; Huang et al., 2011; Lu et al., 2010, 2013; Ng et al., 2009; Sarrach et al., 2018). Induction of EpCAM-dependent proliferation and differentiation has been linked to regulated intramembrane proteolysis (RIP) of the molecule by alpha- and beta-sheddases and the gamma-secretase complex to generate an intracellular signaling moiety termed EpICD (EpCAM intracellular domain). The resulting EpICD domain can translocate into the nucleus and control the transcription of genes with functions in proliferation (cyclin D, c-Myc) and stem cell pluripotency (Oct3/4, Nanog, Sox2, and Klf4) (Chaves-Perez et al., 2013; Hsu et al., 2016; Huang et al., 2011; Kuan et al., 2017; Lu et al., 2010; Maetzel et al., 2009). The functions of EpICD can be controlled at various levels, including its initial cleavage and nuclear translocation (Denzel et al., 2009) and its degradation by the proteasome (Huang et al., 2019).

Orthologs of human EpCAM (hEpCAM) have been identified *in silico* in 52 different species including placental mammals, marsupials, fishes, reptiles, amphibians, and birds (Hachmeister et al., 2013). Sequence homologies suggest that EpCAM is a highly conserved protein throughout the animal kingdom. Accordingly, murine and human EpCAMs are 80% identical at the amino acid level (Bergsagel et al., 1992), and murine EpCAM was also reported to be subject to RIP, with similar cleavage patterns and proteases involved (Hachmeister et al., 2013; Tsaktanis et al., 2015).

During physiological and pathological differentiation in ESCs and in carcinoma cells, respectively, EpCAM exhibits dynamic changes in expression levels and membrane localization. Upon differentiation, ESCs entirely downregulate EpCAM in the majority of cells (Gonzalez et al., 2009; Lu et al., 2010; Ng et al., 2009), a phenomenon equally observed in EpCAM-positive liver progenitor cells upon final differentiation to hepatocytes (Dolle et al., 2014; Schmelzer and Reid, 2008; Schmelzer et al., 2007). This tight regulation results in the formation of cellular patterning with EpCAM-negative mesodermal cells and EpCAM-positive endodermal cells in differentiating ESC and in the developing embryo (Guerra et al., 2012; Lei et al., 2012; McLaughlin et al., 1999, 2001; Nagao et al., 2009; Sarrach et al., 2018). An important function of EpCAM during embryogenesis and tissue morphogenesis lies in the regulation of cell-cell interactions through the modulation of *adherens* junctions and tight junctions and the cortical RhoA zone (Gaston et al., 2021; Guerra et al., 2012; Lei et al., 2012; Salomon et al., 2017). Genetic engineering of ESC demonstrated a requirement for an early spatiotemporal EpCAM patterning, the disturbance of which resulted in impaired differentiation along mesodermal and endodermal lineages (Sarrach et al., 2018). Epigenetic regulation of EpCAM at the transcriptional level was observed in 2D and 3D models of ESC differentiation, early embryonic stages, and in single-cell RNA sequencing of early murine gastrulation (Lu et al., 2010; Sarrach et al., 2018; Scialdone et al., 2016). These regulatory mechanisms include chromatin remodeling and histone modifications in the *EPCAM* promoter (Lu et al., 2010; Sarrach et al., 2018). Additional post-translational regulation of EpCAM availability at the plasma membrane is anticipated, considering a reportedly high protein stability of plasma membrane-localized EpCAM (half-life of 21 h) (Munz et al., 2008) and a delay in mRNA downregulation compared to protein loss (Sarrach et al., 2018). Similarly, in primary tumors and during metastases formation, carcinoma cells are characterized by substantial molecular heterogeneity and undergo phenotypic changes along the epithelial-to-mesenchymal transition (EMT) (Thiery et al., 2009; Thiery and Lim, 2013; Ye and Weinberg, 2015), which are associated with frequent loss of EpCAM in circulating and disseminated tumor cells (Brown et al., 2021; Gires et al., 2020; Gorges et al., 2012; Keller et al., 2019; Liu et al., 2019). Besides transcriptional downregulation and RIP-mediated degradation of the protein at the plasma membrane, endocytosis and lysosomal degradation may account for the loss of EpCAM at the plasma membrane.

In the present study, we have performed a proteomic interactome screen to identify potential binding partners of EpCAM in mouse teratoma cells with the aim to delineate pathways involved in regulation of EpCAM protein dynamics. We describe an association of EpCAM with numerous proteins involved in vesicle and membrane trafficking and demonstrate endocytosis and membrane recycling of EpCAM under physiological conditions during differentiation of ESC and upon EMT induction in carcinoma cells.

## Results

### Identification of murine EpCAM interaction clusters with vesicle transport, mitochondrial, and nuclear transport proteins

**Figure 1:**
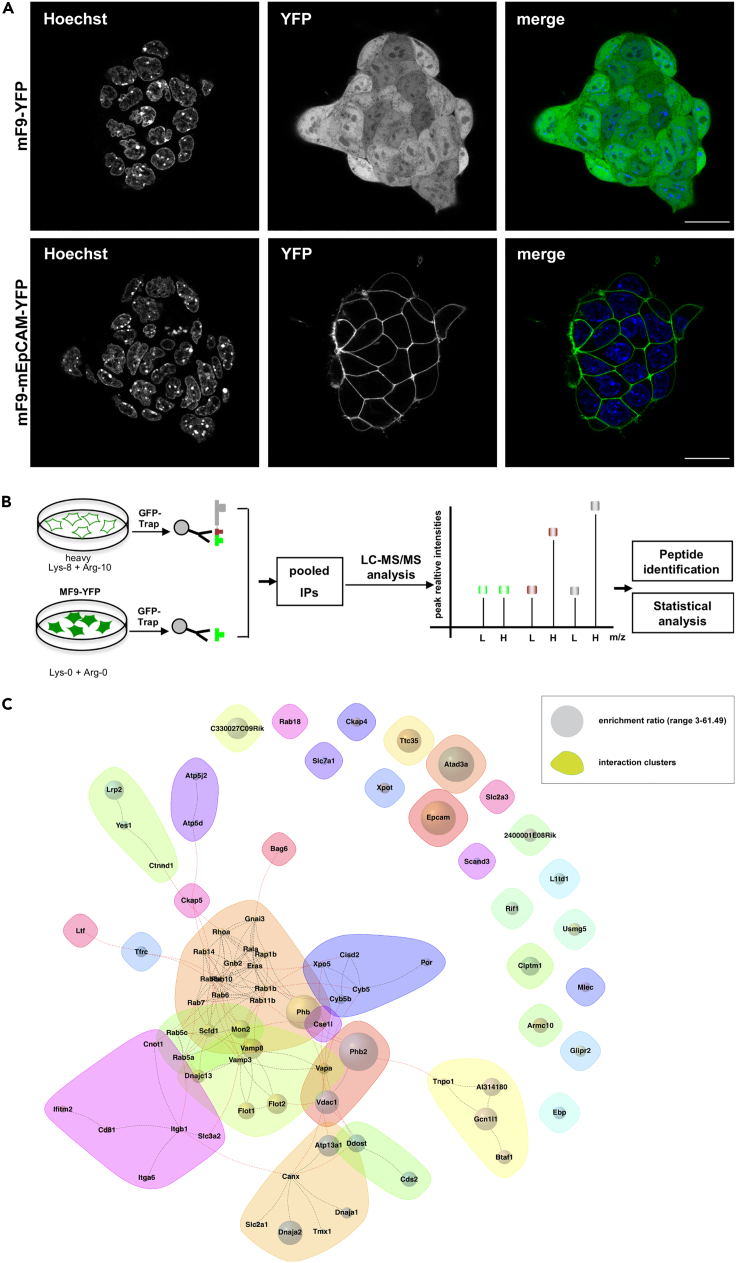
SILAC- and mass spectrometry-based identification of potential interactors of murine EpCAM (A) YFP (upper panels) and EpCAM-YFP (lower panels) were visualized by confocal laser scanning microscopy in stable mF9 cell transfectants expressing YFP (top panels) or EpCAM-YFP (lower panels) in the absence of any additional treatment. Nucleic DNA was visualized with Hoechst 33,342 (left panels). Shown are representative images. Scale bar represents 20 μm. (B) Schematic representation of SILAC screening performed with labeling with heavy (Lys-8/Arg-10) and light amino acids (Lys-0/Arg-0). YFP fusion proteins and interacting proteins were immunoprecipitated with a GFP-trap, pooled, and proteins analyzed upon liquid chromatography and mass spectrometry (LC-MS/MS). (C) Interactome of all known protein-protein interactions between 77 EpCAM partners identified in mF9 cells through SILAC-IP-MS (n = 3; one-tailed t test, permutation-based false discovery rate (FDR) < 0.05) visualized with the STRING database and *R-*package igraph. Hierarchical cluster analysis of the interactome was performed, and interaction clusters were formed based on cluster edge-betweenness. Nodes describe (1) the enrichment ratio (size, 3–61.49); (2) interaction clusters (color); (3) network edges represent known functional interactions in the STRING database.

The murine teratoma cell line mF9 was stably transfected with expression plasmids for murine EpCAM in fusion with yellow fluorescent protein (YFP) at the C-terminus and with YFP, as a control. EpCAM-YFP was correctly localized at the plasma membrane, whereas YFP was homogeneously expressed throughout the cell (Figure 1A). Stable isotope labeling with amino acids in cell culture (SILAC) was performed with EpCAM-YFP and YFP mF9 cell lines upon labeling with ^13^C_6_-^15^N_2_-L-lysin and ^13^C_6_-^15^N_4_-L-arginine (Lys-8 and Arg-10; heavy; H) and normal amino acids (Lys-0 and Arg-0; light; L), respectively. Differentially labeled lysates were subjected to immunoprecipitation of YFP and associated proteins using GFP-Trap® agarose beads before identification through liquid chromatography tandem mass spectrometry (LC-MS/MS) and quantification of enrichment ratios from three biological repeats (Figure 1B). Potential interaction partners of EpCAM-YFP were selected based on (1) >3-fold enrichment versus YFP-associated proteins, (2) at least two unique peptides for quantification, and (3) p values ≤ 0.05 in all n = 3 independent biological repeats. A total of 78 proteins complied with these selection criteria including EpCAM (highest H/L ratio of 61.49; p value = 0.018). Table 1 summarizes all proteins considered significant with Ensemble ID, EpCAM-YFP/YFP ratios (H/L), numbers of unique peptides, gene and protein names, cellular localization, function or protein family, and p values. All additional raw data resulting from the screen are included in Data S1.

**Table 1:** Putative mEpCAM-YFP interacting proteins identified through comparative quantitative SILAC proteomics of mEPCAM-YFP and YFP

Ensembl ID	Mean ratio EpCAM-YFP/YFP	Unique peptides	Gene	Name	Localization	Function/Family	p value
Ensembl: ENSMUSP00000061935	61.49	15	EPCAM	mEpCAM	Cell membrane	Cell adhesion molecule	0.018
Ensembl: ENSMUSP00000004375	30.80	17	PHB2	Prohibitin-2	Mitochondria, cytosol, nucleus	Transcription co-regulator, mitochondrial chaperone	0.01
Ensembl: ENSMUSP00000030903	28.16	15	ATAD3A	ATPase family AAA domain-containing protein 3	Mitochondria	AAA domain ATPase	0.0002
Ensembl: ENSMUSP00000119603	27.45	PHB	PHB1	Prohibitin-1	Cytosol, nucleus	Transcription co-regulator	0.0045
Ensembl: ENSMUSP00000034138	19.48	14	DNAJA2	DnaJ homolog subfamily A member 2	Cytosol, membrane	Co-chaperone of Hsc70	0.012
Ensembl: ENSMUSP00000022962	19.17	4	TTC35	ER membrane protein complex subunit 2 (Emc2)	ER, cytosol, nucleus, mitochondria	ERAD, ER-mitochondria tethering	0.034
Ensembl: ENSMUSP00000020673	18.91	19	VDAC1	Voltage-dependent anion-selective channel 1	Outer mitochondrial/cell membranes	Ion channel	0.032
Ensembl: ENSMUSP00000069432	18.25	61	GCN1	eIF-2-alpha kinase activator	Cytosol, membrane	Chaperone of uncharged tRNAs	0.017
Ensembl: ENSMUSP00000034326	17.20	8	ATP13A1	Manganese-transporting ATPase 13A1	ER membrane	Cation-transporter	0.025
Ensembl: ENSMUSP00000044714	17.15	9	C330027C09	Cancerous inhibitor of PP2A	Membrane, cytosol	Cadherin-mediated cell adhesion	0.024
Ensembl: ENSMUSP00000059501	16.41	3	VAMP8	Vesicle-associated membrane protein 8	Lysosome/endosome/cell membranes	SNARE involved in autophagy	0.027
Ensembl: ENSMUSP00000098349	16.14	6	FLOT2	Flotillin-2	Cell membrane, endosome	Formation of caveolae	0.029
Ensembl: ENSMUSP00000079752	15.36	6	LRP22	Low-density lipoprotein receptor-related 2	Membrane	HDL endocytosis	0.016
Ensembl: ENSMUSP00000051293	15.09	6	CLPTM1	Cleft lip and palate transmembrane protein 1	Membrane	T cell differentiation	0.013
Ensembl: ENSMUSP00000073462	14.61	5	MON2	Mon2	Cytosol	Golgi-ER trafficking	0.038
Ensembl: ENSMUSP00000036198	14.53	21	AI314180	Proteasome-associated protein ECM29 homolog	Cytosol	Proteasome assembly	0.005
Ensembl: ENSMUSP00000030538	14.28	8	DDOST	Dolichyl-diphosphooligosaccharide protein glycosyltransferase 48kD subunit	ER	Essential subunit of the N-oligosaccharyl transferase complex	0.044
Ensembl: ENSMUSP00000001569	13.62	6	FLOT1	Flotillin-1	Cell membrane, endosome	Formation of caveolae	0.008
Ensembl: ENSMUSP00000099470	12.25	2	CDS2	Phosphatidate cytidylyltransferase 2	Mitochondrial inner membrane	CDP-diacyglycerol provider	0.043
Ensembl: ENSMUSP00000072669	12.08	3	ARMC10	Armadillo repeat-containing protein 10	ER	Suppressor of p53 transcriptional activity	0.005
Ensembl: ENSMUSP00000097093	11.26	3	BTAF1	TATA-binding protein-associated factor 172	Nucleus	ATPase	0.026
Ensembl: ENSMUSP00000033131	10.72	3	2400001E08Rik	Ragulator complex protein LAMTOR1	Endo/lysosome, cell membrane	Amino acid sensing and mTORC1 activation	0.024
Ensembl: ENSMUSP00000055206	10.54	14	DNAJC13	DnaJ heat shock protein family (Hsp40) member C13	Cytosol, endosome, lysosome	Chaperone, endosome organization	0.021
Ensembl: ENSMUSP00000064155	10.53	11	RIF1	Telomere-associated protein Rif1	Nucleus	DNA damage checkpoint	0.032
Ensembl: ENSMUSP00000021335	10.20	4	SCFD1	Sec1 family domain-containing protein 1	Cytosol, ER membrane, Golgi apparatus	SNARE-pin assembly, ER transport	0.03
Ensembl: ENSMUSP00000093713	10.16	2	USMG5	Up-regulated during skeletal muscle growth protein 5	Mitochondrial membrane	Maintenance of ATP synthase in mitochondria	0.00015
Ensembl: ENSMUSP00000033509	10.00	2	EBP	Emopamil binding protein	ER and nuclear membrane	Sterol isomerase	0.032
Ensembl: ENSMUSP00000127504	9.23	23	L1TD1	LINE-1 type transposase domain-containing protein 1	Cytosol	RNA-binding	0.045
Ensembl: ENSMUSP00000030202	9.11	5	GLIPR2	Golgi-associated plant pathogenesis-related protein 2	Golgi apparatus membrane	EMT, ERK regulation, negative regulator of autophagy	0.00045
Ensembl: ENSMUSP00000030118	8.86	17	DNAJA1	DnaJ homolog subfamily A member 1	Cytosol, membrane	Co-chaperone of Hsc70	0.0058
Ensembl: ENSMUSP00000024897	8.82	9	VAPA	Vesicle-associated membrane protein-associated protein A	ER, cell membrane	Activation of RRas signaling, ER morphology and vesicle trafficking	0.029
Ensembl: ENSMUSP00000023486	8.77	18	TFRC	Transferrin receptor protein 1	Cell membrane	Iron uptake	0.028
Ensembl: ENSMUSP00000043488	8.64	9	XPOT	Exportin-T	Cytosol, nucleus	Nuclear export of tRNAs	0.015
Ensembl: ENSMUSP00000055776	7.96	5	MLEC	Malectin	ER membrane	N-glycosylation, Glc2-N-glycan binding protein	0.04
Ensembl: ENSMUSP00000034400	7.57	6	CYB5B	Cytochrome b5 type B	Mitochondrion outer membrane	Electron carrier	0.018
Ensembl: ENSMUSP00000030797	7.34	5	VAMP3	Vesicle-associated membrane protein 3	Lysosome/endosome/cell membranes	SNARE involved in endosome to *trans*-Golgi network	0.01
Ensembl: ENSMUSP00000050336	7.34	11	CKAP4	Cytoskeleton-associated protein 4	Cytosol, ER, cell membrane	Anchoring of ER to microtubules Dickkopf1 receptor involved in tumor progression	0.031
Ensembl: ENSMUSP00000072154	7.17	2	YES1	Tyrosine protein kinase Yes	Cell membrane, cytosol	Non-receptor tyrosine kinase involved in cell growth, survival, apoptosis, cell-cell adhesion and differentiation	0.0005
Ensembl: ENSMUSP00000046714	6.66	4	SLC7A1	High affinity cationic amino acid transporter 1	Cell membrane	Amino acid transport	0.044
Ensembl: ENSMUSP00000005651	6.53	6	POR	NADPH-cytochrome p450 reductase	ER membrane	Electron transfer from NADP to cyt P450	0.019
Ensembl: ENSMUSP00000125504	6.36	3	ATP5J2	ATP synthase subunit f	Mitochondrion	Mitochondrial membrane ATP synthase	0.009
Ensembl: ENSMUSP00000002790	6.10	37	CSE1L	Exportin-2	Nucleus, cytosol	Export receptor for importin-alpha	0.014
Ensembl: ENSMUSP00000044533	6.01	5	SCAND3	Scan domain-containing protein 3	Mitochondria, nucleoplasm	Unknown	0.015
Ensembl: ENSMUSP00000066238	5.94	2	RAP1B	Ras-related protein 1b	Cell membrane, cytosol	GTPase involved in endothelial cell polarity and barrier function	0.0015
Ensembl: ENSMUSP00000007959	5.86	2	RHOA	Transforming protein RhoA	Cell membrane, cytosol	Focal adhesion assembly and signaling	0.019
Ensembl: ENSMUSP00000025804	5.74	5	RAB1B	Ras-related protein Rab-1B	ER, Golgi apparatus, mitochondria	Intracellular membrane trafficking and vesicular transport between ER and Golgi	0.01
Ensembl: ENSMUSP00000032946	5.72	5	RAB6A	Ras-related protein Rab-6A	Golgi apparatus	Intracellular membrane trafficking from Golgi to ER	0.028
Ensembl: ENSMUSP00000028238	5.64	8	RAB14	Ras-related protein Rab-14	Endosome, Golgi apparatus	Intracellular membrane trafficking from Golgi to ER Regulation of endocytic transport of ADAM10, N-cadherin/CHD2 shedding and cell-cell adhesion	0.02
Ensembl: ENSMUSP00000043508	5.62	15	TNPO1	Transportin 1	Cytosol, nucleus	Nuclear protein import	0.031
Ensembl: ENSMUSP00000071470	5.51	2	IFITM2	Interferon-induced transmembrane protein 2	Cell membrane	Antiviral protein Induces cell cycle arrest and induction of apoptosis	0.0005
Ensembl: ENSMUSP00000043768	5.49	3	CD81	CD81; Target of antiproliferative antibody 1 (TAPA-1)	Cell membrane	Regulation of lymphoma cell growth	0.0064
Ensembl: ENSMUSP00000084257	5.47	14	XPO5	Exportin-5	Nucleus, cytosol	Nuclear export double-stranded RNA binding proteins and double-stranded RNAs	0.014
Ensembl: ENSMUSP00000092658	4.98	14	RAB7	Ras-related protein Rab-7	Golgi apparatus, endosome	Regulation of endo-lysosomal trafficking	0.036
Ensembl: ENSMUSP00000003156	4.96	2	ATP5D	ATP synthase, H+ transporting, mitochondrial F1 complex, delta subunit, isoform CRA_c	Mitochondria	Proton-transporting ATP synthase	0.0085
Ensembl: ENSMUSP00000019317	4.88	3	RAB5C	Ras-related protein Rab-5c	Endosome	Intracellular membrane trafficking	0.029
Ensembl: ENSMUSP00000095285	4.71	6	RAB18	Ras-related protein Rab18	Cell membrane	Endocytosis and recycling of proteins	0.047
Ensembl: ENSMUSP00000020637	4.68	18	CANX	Calnexin	ER	Protein assembly and ER-retention of incorrectly folded proteins	0.02
Ensembl: ENSMUSP00000110021	4.63	11	RAB11B	Ras-related protein Rab11b	Recycling endosome membrane	Intracellular membrane trafficking and endocytic recycling	0.0175
Ensembl: ENSMUSP00000021001	4.59	2	RAB10	Ras-related protein Rab10	Golgi apparatus, endosome, cytoplasmic vesicles	Intracellular trafficking from Golgi to cell membrane	0.003
Ensembl: ENSMUSP00000106656	4.55	2	GNB2	Guanine nucleotide-binding protein subunit 2	Cytosol	G-protein coupled signaling	0.0175
Ensembl: ENSMUSP00000107729	4.47	11	ITGA6	Integrin alpha-6	Cell membrane	Receptor for laminin in epithelial cells Essential for NRG1-ERBB and IGF1 signaling	0.03
Ensembl: ENSMUSP00000030398	4.37	7	SLC2A1	Solute carrier family 2, facilitated glucose transporter member 1	Cell membrane	Glucose transporter	0.015
Ensembl: ENSMUSP00000097303	4.37	13	CKAP5	Cytoskeleton-associated protein 5	Cytosol	Regulation of microtubule dynamics	0.018
Ensembl: ENSMUSP00000029815	4.18	5	CISD2	CDGSH iron-sulfur domain-containing protein 2	ER, mitochondria	Regulation of autophagy at the ER	0.0074
Ensembl: ENSMUSP00000130194	4.17	17	SLC3A2	Solute carrier family 3 member 2	Cell membrane	Amino acid transport to the membrane	0.012
Ensembl: ENSMUSP00000124480	4.17	4	CYB5	Cytochrome b5	ER	Electron carrier	0.0085
Ensembl: ENSMUSP00000107323	4.13	4	CTNND1	Catenin delta-1	Cytosol, nucleus, cell membrane	WNT signaling Cadherin-mediated adhesion EGF-R/PDGF-R/CSF-1R/ERBB2 signaling	0.0289
Ensembl: ENSMUSP00000009003	4.13	3	RALA	Ras-related protein Ral-a	Cell membrane	Multifunctional GTPase involved in GTP-dependent exocytosis and signaling	0.0013
Ensembl: ENSMUSP00000017975	4.13	3	RAB5A	Ras-related protein Rab-5a	Cell membrane, endosome, cytosol	Intracellular trafficking from cell membrane to early endosomes	0.036
Ensembl: ENSMUSP00000021471	4.12	2	TMX1	Thioredoxin-related transmembrane protein 1	ER membrane	Redox-reaction	0.001
Ensembl: ENSMUSP00000033500	4.12	6	ERAS	Embryonic Ras	Cell membrane	GTPase involved in embryonic stem cell teratogenesis	0.0289
Ensembl: ENSMUSP00000096232	4.08	4	RAB8A	Ras-related protein Rab8a	Cell membrane, Golgi apparatus, endosome	Intracellular trafficking, exocytosis, polarized vesicular trafficking	0.043
Ensembl: ENSMUSP00000087457	4.04	9	ITGB1	Integrin beta-1	Cell membrane	Integrin-mediated adhesion Interacts with Integrin alpha-6 as a receptor for laminin	0.0039
Ensembl: ENSMUSP00000000001	3.85	3	GNAI3	Guanine nucleotide-binding protein G 8K) subunit alpha	Cytosol, cell membrane	G-protein coupled signaling	0.029
Ensembl: ENSMUSP00000032476	3.59	10	SLC2A3	Solute carrier family 2, facilitated glucose transporter member 3	Cell membrane	Glucose transporter	0.0038
Ensembl: ENSMUSP00000127808	3.24	7	RP24-421P3.2	Unknown predicted protein	unknown	unknown	0.012
Ensembl: ENSMUSP00000096073	3.24	28	CNOT1	CCR4-NOT transcription complex subunit 1	Cytosol, P-body, nucleus	mRNA deadenylation	0.034
Ensembl: ENSMUSP00000025250	3.06	8	BAG6	Bcl2-associated anthanogene 6	Cytosol, nucleus	Chaperone	0.0039

Criteria: EpCAM-YFP/YFP ratio >3, p value < 0.05.

Interaction clusters within the interactome were analyzed using the Search Tool for the Retrieval of Interacting Genes/Proteins (STRING) database and the *R*-package *igraph* and were formed based on edge-betweeness clustering. This revealed major clusters associated with the Ras-superfamily small G-proteins of the Rab family, flotillins, integrins alpha α2 and β1 (collagen 1 receptor), prohibitins 1 and 2, and nuclear import/export proteins Xpo5 and CSE1L (Figure 1C).

**Figure 2:**
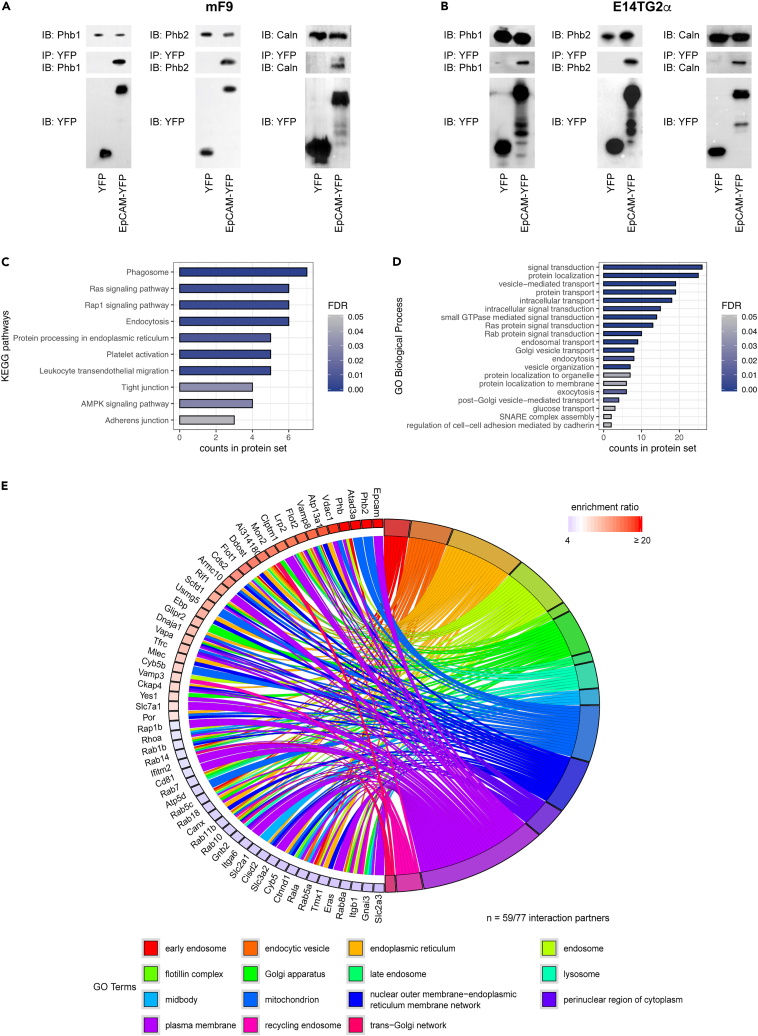
Classification of potential interactors of EpCAM (A and B) Two potential interactors of EpCAM with high enrichment scores (Prohibitin 1 and 2 Phb1 and Phb2) and one potential interactor with lower enrichment score (Calnexin, Caln) were co-precipitated with EpCAM-YFP but not YFP in stable transfectants of mF9 teratocarcinoma cells (A) and in E14TG2α murine embryonic stem cells (B). Phb1, Phb2, and Caln levels were comparable in whole-cell lysates of EpCAM-YFP and YFP stable transfectants of mF9 and E14TG2α. Amounts of EpCAM-YFP and YFP following immunoprecipitation with GFP-Trap® agarose beads were controlled with GFP-specific antibodies and were comparable. Shown are each one representative immunoblot from three independent experiments. (C and D) KEGG Pathways and Gene Ontology (GO) terms “Biological process” (BP) to analyze potential interactors of EpCAM. KEGG pathways and GO terms are depicted with the protein counts in each set and false-discovery rate (FDR) ≤ 0.05. (E) Potential interactors of EpCAM (n = 59/77) were implemented on the left side of a chord diagram with their respective enrichment ratio and the GO terms in which they feed into on the right side of the chord diagram.

To validate results from the SILAC screen, we selected high- and low-ranking interactors for confirmatory Co-IPs in mF9 cell lysates. Interactions of EpCAM-YFP with prohibitin 1 and 2, which were characterized by high enrichments scores (27.45 and 30.80, respectively), and calnexin, which was characterized by a lower enrichment score (4.68), were assessed. Co-precipitation of prohibitin 1, prohibitin 2, and calnexin with EpCAM-YFP but not YFP confirmed protein-protein interactions of EpCAM-YFP with two top-ranking and one low-ranking potential interactors (Figure 2A). Since EpCAM is also strongly expressed in pluripotent ESCs (Gonzalez et al., 2009), interaction of EpCAM-YFP with prohibitin 1 and 2 and calnexin was assessed, and enrichment was confirmed in E14TG2α ESCs (Figure 2B).

Classifications according to Kyoto Encyclopedia of Genes and Genomes (KEGG) pathways and gene ontology (GO) terms “*Biological processes*”, “*Cellular components*”, and “*Molecular functions*” were conducted to categorize all n = 77 potential interactors of EpCAM identified via SILAC-MS (Tables S1–S3). KEGG pathways and GO terms with the highest protein counts and false discovery rates (FDRs) below 0.05 were related to membrane protein trafficking, endoplasmic reticulum and Golgi apparatus, and mitochondria. The identified terms comprised potential interactors such as flotillin, VAMP8, VAPA and B and numerous Rab proteins that are central effector molecules in endocytosis (Figures 2C and 2D). Potential interactors (n = 59/77) including EpCAM were ranked according to their enrichment scores and implemented in a chord diagram showing the top 15 GO terms in “Cellular Components”, into which the potential interactors feed. GO terms confirmed the involvement of potential EpCAM interactors in protein trafficking and endocytosis processes, endoplasmic reticulum, Golgi apparatus, and mitochondria (Figure 2E).

### EpCAM is present in acidified intracellular vesicles

Three major functional areas were identified from the classification of potential EpCAM interactors: interactions with components of the endoplasmatic reticulum, mitochondria, and vesicle-mediated intracellular transport. As EpCAM is a transmembrane protein, an association with components of the anterograde transport including the endoplasmatic reticulum is expected and was not further addressed. Prohibitin 1 and 2 are integral components of the inner mitochondrial membrane, suggesting a potential localization and function of EpCAM in mitochondria. Biochemical and imaging-based assessment of a possible localization of EpCAM in or at mitochondria was inconclusive and was therefore discontinued.

**Figure 3:**
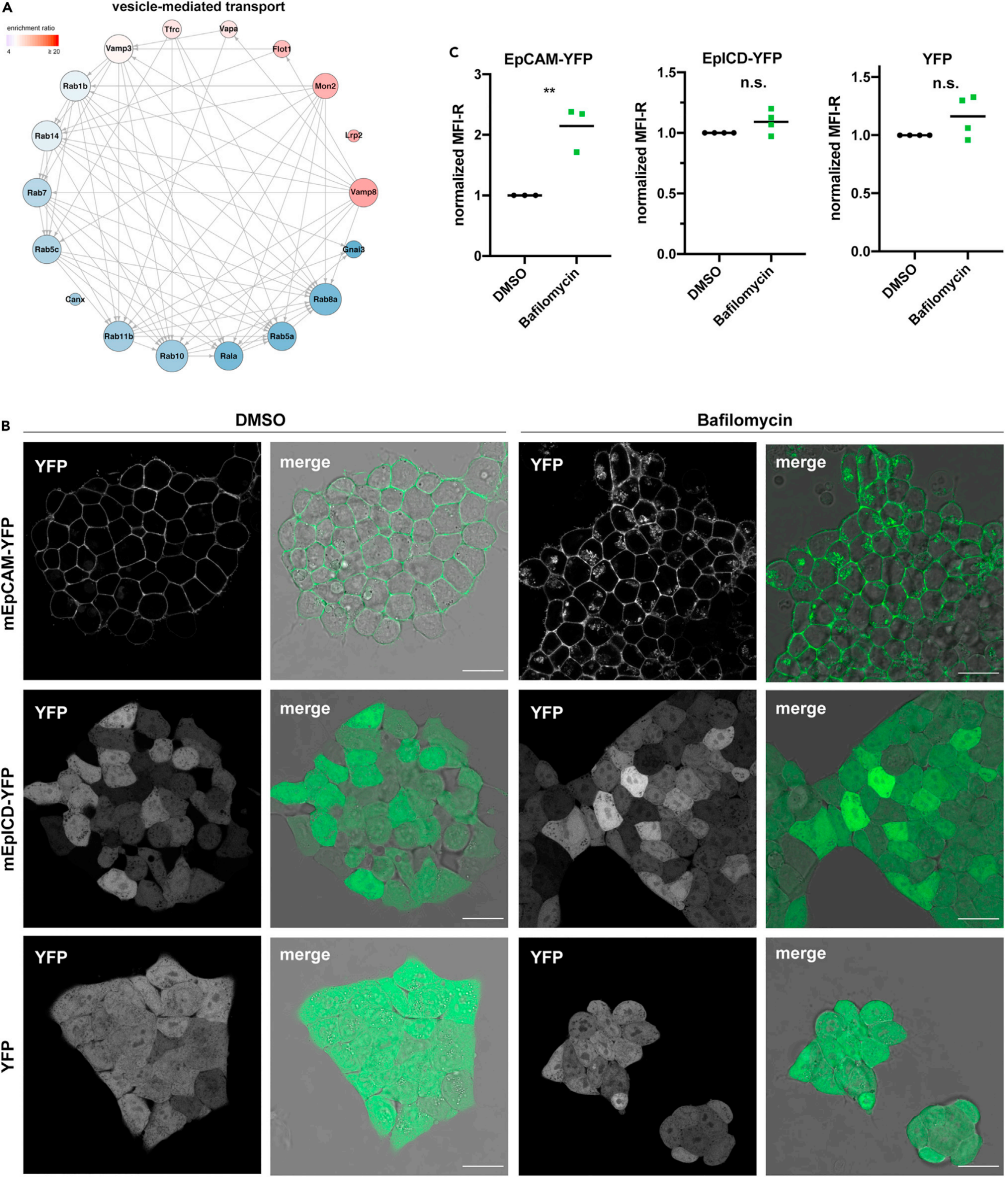
EpCAM localizes to intracellular vesicles (A) Spherical network of SILAC potential interactors of EpCAM found in the GO BP term “vesicle-mediated transport”. Degree of interactions encodes node size. Color shows the enrichment ratio from low (blue) to high (red). Arrow indicates direction of interaction. (B) mF9 cells stably transfected with EpCAM-YFP, mEpICD-YFP, or YFP were treated with DMSO or the V-ATPase inhibitor bafilomycin A1 (10 nM). Bright field and YFP fluorescence were visualized by confocal laser scanning microscopy. Shown are representative pictures. Scale bars represents 20 μm. (C) mF9 cells stably transfected with EpCAM-YFP, EpICD-YFP, or YFP were treated with DMSO or bafilomycin A1. Mean fluorescence intensity ratios were assessed by flow cytometry. Shown are the results from 3 to 4 independent experiments. Mean values are indicated by a line. p value: ∗∗ ≤0.01, n.s.: not significant.

Potential EpCAM interactors that are specifically involved in vesicle-mediated transport and are components of an interaction network are depicted in Figure 3A. Rab proteins 1b, 5a, 5c, 7, 8a, 10, 11b, and 14 were part of this interaction network and have central roles in intracellular transport of cargo molecules. Based on the dynamic spatiotemporal regulation of EpCAM expression at the plasma membrane under normal and pathological conditions, we further concentrated on a retrograde transport of EpCAM and degradation in intracellular vesicles. In order to investigate internalization of EpCAM, mF9 cells stably transfected with EpCAM-YFP were treated with bafilomycin A1, an inhibitor of acidification and protein degradation in lysosomes (Yoshimori et al., 1991). Thereby, a potential endocytosis of EpCAM can occur but further degradation in lysosomes is prevented. Additionally, Bafilomycin A1 has been reported to slowdown the recycling of certain receptors, which may further help visualizing EpCAM protein in intracellular vesicles (Johnson et al., 1993). Treatment with bafilomycin A1 (10 nM) resulted in accumulation of EpCAM-YFP in intracellular vesicles suggesting that EpCAM is endocytosed and degraded in acidic intracellular vesicles (Figure 3B). As described earlier, EpCAM is post-translationally processed via RIP yielding a soluble intracellular domain EpICD via the intermediate of a membrane-tethered C-terminal fragment (Maetzel et al., 2009) (see scheme in Figure S1A). EpICD-YFP was stably expressed in mF9 cells and localized in the cytoplasm and nucleus of control-treated cells (DMSO; Figure 3B). Neither the treatment of EpICD-YFP nor of control YFP-expressing cells resulted in any apparent accumulation in intracellular vesicles (Figure 3B). Next, a pre-cleaved version of EpCAM composed of a 15-amino acids short membrane-proximal portion of the extracellular domain, the transmembrane domain, and the intracellular domain fused to YFP corresponding to the EpCAM C-terminal fragment (mEp-CTF; Figure S1A) was stably expressed in mF9 cells. Cleavage of mEp-CTF by the gamma-secretase complex was inhibited using DAPT, resulting in a stabilization of mEp-CTF at the plasma membrane (Huang et al., 2019). Additional treatment of the cells with bafilomycin A1 resulted in an accumulation of mEp-CTF in intracellular vesicles, demonstrating that membrane tethering is a prerequisite for the endocytosis and degradation of EpCAM in acidified vesicles and suggesting that the internalization motif of EpCAM is located within its C-terminal fragment (Figure S1B).

Quantification of YFP fluorescence by flow cytometry, as a measure of the expression level of EpCAM-YFP, Ep-CTF-YFP, EpICD-YFP, or YFP, exhibited a significant 2.14-fold increase in YFP intensities after bafilomycin A1 treatment of EpCAM-YFP cells but no effect on EpICD-YFP and YFP expression levels (Figure 3C). Quantification of YFP intensities of Ep-CTF-YFP cells showed a 2.8-fold increase of DAPT and bafilomycin A1-treated cells over DAPT-treated cells (Figure S1C). Hence, from these experiments, we conclude that inhibition of vesicle acidification using bafilomycin A1 allowed for the accumulation and detection of EpCAM-YFP molecules following retrograde transport into intracellular vesicles.

### Endocytosis and membrane recycling of EpCAM

**Figure 4:**
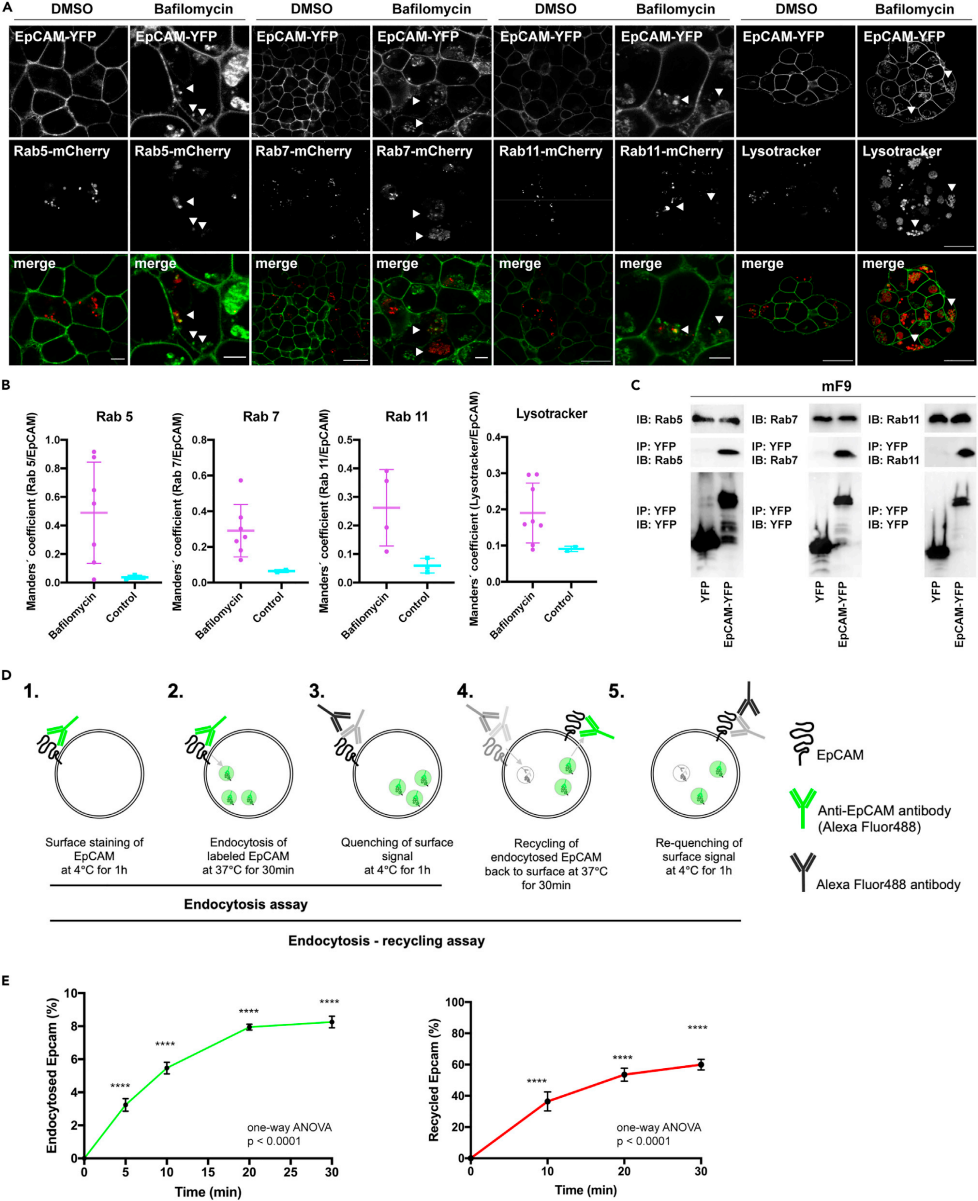
Endocytosis of EpCAM in mF9 teratoma cells (A) mF9 cells stably transfected with EpCAM-YFP and transiently transfected with m-Cherry-tagged Rab5, Rab7, and Rab11 were visualized by laser scanning confocal microscopy. Lysosomes were detected with Lysotracker (Red DND-99 Ex577/Em590 nm). Cells were treated with either DMSO or 10 nM bafilomycin A1 (10 nM) as indicated. Scale bars represent: Rab5 5 μm; Rab7 and Rab11 20 μm (DMSO) and 5 μm (Bafilomycin); Lysotracker: 20 μm. Brightness and contrast of both channels were adjusted linearly. (B) Co-localizations of EpCAM-YFP with the indicated mCherry-tagged Rab proteins and lysosomes were assessed as indicated in STAR Methods. Shown are Manders' coefficients representing the fraction of mCherry-tagged Rab protein or lysotracker overlapping with EpCAM-YfP as dot plots with mean and SD from each n = 7 and n = 2–3 independent imaging areas for bafilomycin A1-treated cells and DMSO-treated cells, respectively. (C) Whole-cell lysates from mF9 cells stably transfected with EpCAM-YFP or YFP were immunoprecipitated with GFP-Trap® agarose beads. Immunoprecipitated proteins were separated by SDS-PAGE and detected with anti-YFP, anti-Rab5, anti-Rab7, and anti-Rab11 antibodies in combination with HRP-conjugated secondary antibodies. Additionally, whole-cell lysates were separated by SDS-PAGE, and Rab5, Rab7, and Rab11 proteins were detected with specific antibodies in combination with HRP-conjugated secondary antibodies. Shown are representative results from three independent experiments. (D) Schematic representation of the endocytosis and membrane recycling assay. (E) Kinetics of EpCAM endocytosis (left panel) and membrane recycling (right panel). Shown are mean percentages of EpCAM endocytosis and membrane recycling with SD over a time of 30 min from three independent experiments. One-way ANOVA with Dunnet's multiple tests. ∗∗∗∗ p value < 0.0001.

To assess the presence of EpCAM in early, late, and recycling endosomes, EpCAM-YFP-expressing mF9 cells were transiently transfected with mCherry-tagged versions of Rab5, Rab7, and Rab11. Localization of EpCAM-YFP within intracellular vesicles was confirmed by laser scanning confocal fluorescence microscopy (Figure 4A). Localization of EpCAM-YFP in lysosomes/acidic compartments was confirmed by co-staining with lysotracker (Figures 4A and 4B). Co-localization of EpCAM-YFP with mCherry-tagged versions of Rab5, Rab7, and Rab11 and with lysosomes was quantified using Manders' coefficients in bafilomycin A1-treated mF9 cells compared to DMSO-treated cells. Accumulation of EpCAM-YFP in intracellular vesicles upon bafilomycin A1 treatment was associated with increased Manders' coefficients, representing an increased fraction of all three Rab proteins and lysosomes overlapping with EpCAM-YFP (Figure 4B). Co-localization of EpCAM with Rab5, Rab7, and Rab11 was confirmed by immunofluorescence staining of endogenous proteins (Figure S2). Interaction of EpCAM with Rab5, Rab7 and Rab11 was further investigated through co-immunoprecipitation experiments in stably EpCAM-YFP transfected mF9 cells. EpCAM-YFP but not YFP co-precipitated with Rab5, Rab7, and Rab11 following immunoprecipitations of whole-cell lysates with GFP-Trap® agarose beads (Figure 4C).

Endocytosis of endogenous EpCAM in untreated mF9 cells was addressed with an antibody-based internalization and membrane recycling assay (see STAR Methods). The assay relies on labeling of EpCAM on the plasma membrane with an EpCAM Alexa 488-conjugated antibody and subsequent quenching of the remaining cell surface fluorescence after internalization of EpCAM with an anti-Alexa 488 antibody (Figure 4D, steps 1–3). Samples were assessed every 5 min and demonstrated increasing endocytosis of EpCAM that reached a plateau at approx. 20 min with a mean percentage of endocytosed molecules of 8.25% (Figure 4E, left panel). Additionally, membrane localization of stained endogenous EpCAM, quenching, and endocytosis were confirmed by confocal imaging in samples of mF9 cells subjected to the internalization assay (Figure S3A). Membrane recycling of EpCAM was assessed by allowing cells to recycle EpCAM to the membrane after initial internalization, followed by quenching of the remaining cell surface fluorescence with anti-Alexa 488 antibody. The amount of recycled EpCAM was calculated as a percentage of endocytosed EpCAM (Figure 4D, steps 4 and 5). A recycling rate of 59.93% was determined for endogenous EpCAM in mF9 cells (Figure 4E, right panel). Hence, EpCAM localizes in Rab5-, Rab7-, and Rab11-associated early, late, and recycling endosomes and is subject to endocytosis and membrane recycling.

### Enhanced EpCAM endocytosis in mesodermal differentiation of ESC and EMT of carcinoma cells

The expression of EpCAM is tightly regulated and relates to the tissue of origin. Both, *in vitro* and *in vivo*, EpCAM expression is repressed in pluripotent ESCs undergoing mesodermal differentiation while it is retained in endodermal tissue (Sarrach et al., 2018). In mouse embryonic development, loss of EpCAM is observed at the single-cell level during initial steps of gastrulation with the emergence of EpCAM^low/negative^ early mesodermal progenitors (Sarrach et al., 2018; Scialdone et al., 2016).

**Figure 5:**
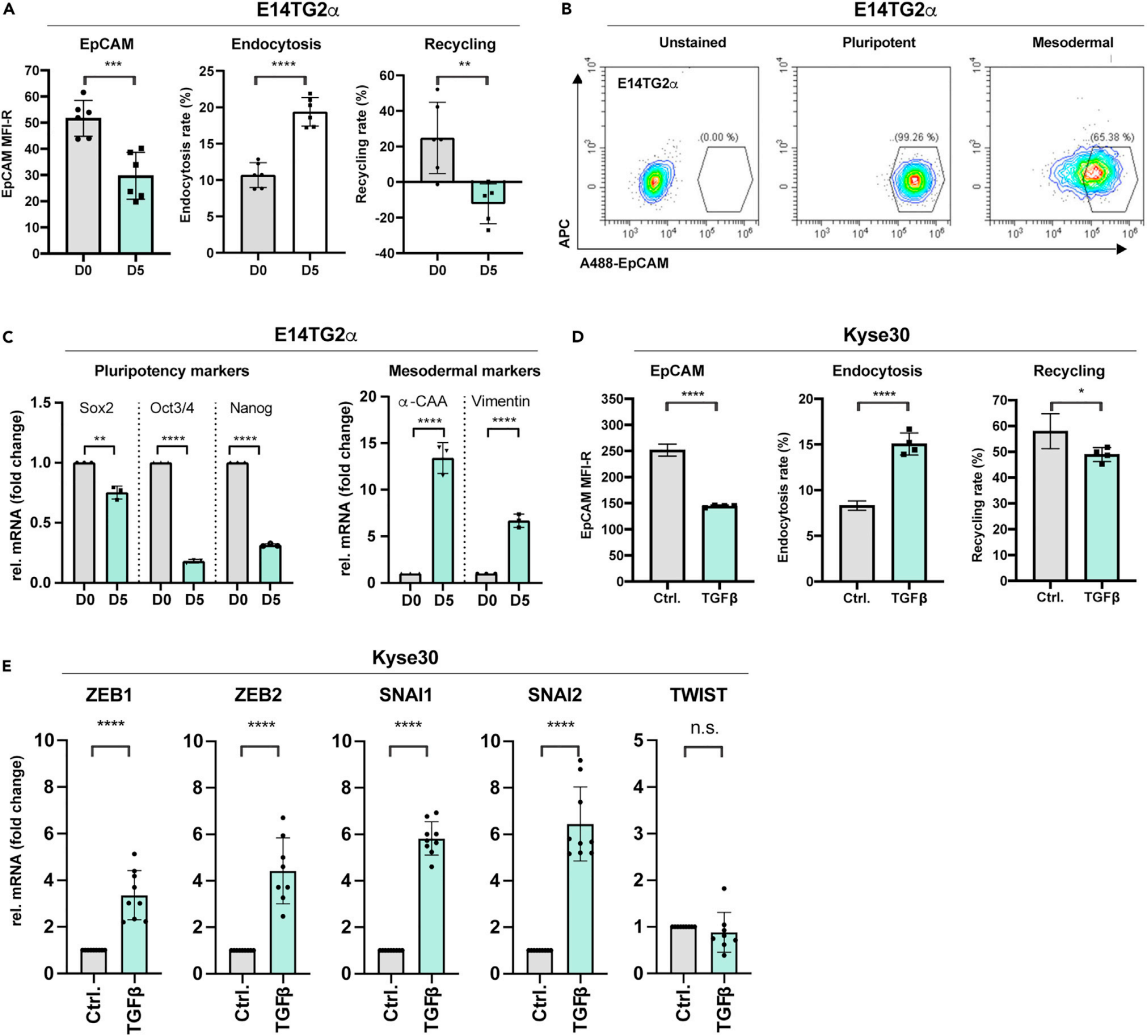
Endocytosis of EpCAM during mesodermal differentiation of ESC (A) Left: EpCAM expression in E14TG2α ESC under pluripotency (day 0, D0) and following mesodermal differentiation (see STAR Methods) was analyzed with Alexa-488-labeled specific antibody. Shown are scatter dot plots with means and SD of n = 3 independent experiments performed in duplicates. Middle and right: EpCAM endocytosis (middle) and membrane recycling (right) was assessed in E14TG2α ESC under pluripotency (day 0, D0) and following mesodermal differentiation. Shown are scatter dot plots with means and SD of n = 3 independent experiments performed in duplicates. Student's t test; ∗∗ 0.01, ∗∗∗ 0.001, ∗∗∗∗ 0.0001. (B) EpCAM expression in E14TG2α ESC under pluripotency (day 0, D0) and following mesodermal differentiation was analyzed with Alexa-488-labeled specific antibody. Shown are representative examples of unstained pluripotent ESCs and stained pluripotent (D0) and mesodermally differentiated ESCs (D5) in gated dot plots from n = 3 independent experiments performed in duplicates. (C) Expression of pluripotency markers Sox2, Oct3/4 and Nanog, and mesodermal markers α-CAA and vimentin was quantified by qRT-PCR in E14TG2α ESC under pluripotency (day 0, D0) and following mesodermal differentiation. Shown are scatter dot plots with means and SD of n = 3 independent experiments performed in triplicates. Student's t test is indicated. ∗∗ ≤0.01; ∗∗∗∗ ≤0.0001. (D) Left: EpCAM expression in Kyse30 carcinoma cells under control (Ctrl.) and following TGFβ treatment (TGFβ) (see STAR Methods) was analyzed with Alexa-488-labeled specific antibody. Shown are scatter dot plots with means and SD of n = 3 independent experiments performed in duplicates. Middle and right: EpCAM endocytosis (middle) and membrane recycling (right) was assessed in Kyse30 cells under control (Ctrl.) and following TGFβ treatment (TGFβ). Shown are scatter dot plots with means and SD of n = 3 independent experiments performed in duplicates. Student's t test; ∗∗ 0.01, ∗∗∗∗ 0.0001. (E) The mRNA expression of EMT transcription factors ZEB1/2, SNAI1/2, and TWIST was quantified by qRT-PCR in Kyse30 cells under control (Ctrl.) and following TGFβ treatment (TGFβ). Shown are scatter dot plots with means and SD of n = 3 independent experiments performed in triplicates. Student's t test; ∗∗∗∗ 0.0001, n.s. not significant.

To address whether endocytosis is instrumental in the withdrawal of EpCAM from the plasma membrane in differentiating cells, murine pluripotent ESCs were subjected to a guided mesodermal differentiation protocol (See STAR Methods). EpCAM expression at the plasma membrane was analyzed by flow cytometry under pluripotency (day 0) and upon finalization of the mesodermal differentiation protocol (day 5). EpCAM expression at the plasma membrane was reduced to 57.16% on average, which translates to a reduction in mean fluorescence intensity ratio from an average of 51.68 to 29.72 (Figure 5A, left panel and Figure 5B). In parallel, internalization and membrane recycling of EpCAM were monitored according to the protocol depicted in Figure 4C. Membrane localization, quenching, and endocytosis were confirmed by confocal imaging in E14TG2α ESC (Figure S3B). Upon guided mesodermal differentiation, endocytosis of EpCAM increased from an average of 10.68% under pluripotency to 19.38% following mesodermal differentiation (Figure 5A, middle panel). Recycling of EpCAM to the plasma membrane was entirely blocked from 24.81% under pluripotency to −12.21% following mesodermal differentiation (Figure 5D, right panel). Negative recycling values most likely reflected residual ongoing internalization of EpCAM during recycling steps. Mesodermal differentiation was confirmed by the loss of pluripotency markers Sox2, Oct3/4, and Nanog and the induction of the cardiomyocyte marker α-cardiac actin (α-CAA) and the mesodermal marker vimentin (Figure 5C).

Next, EMT was induced in the EpCAM-positive esophageal carcinoma cell line Kyse30 upon treatment with TGFβ. EpCAM expression was reduced following TGFβ treatment with the MFI-R decreasing from an average of 251.75 to 144.25 (Figure 5D, left panel). In parallel, endocytosis rates were increased from an average of 8.3%–15.05%, and recycling rates were moderately decreased from an average of 57.98%–48.9% (Figure 5D, middle and right panels). EMT induction via TGFβ was confirmed by the enhanced expression of EMT transcription factors ZEB1, ZEB2, SNAI1 (Snail), and SNAI2 (Slug) (Figure 5E).

Hence, EpCAM downregulation during mesodermal differentiation in ESCs and during EMT in carcinoma cells is linked to enhanced endocytosis and a block or reduction of its recycling to the plasma membrane.

## Discussion

EpCAM displays tissue selectivity with dynamic changes in expression strength and patterning. Spatiotemporal cell-specific expression of EpCAM is best exemplified in differentiating ESCs and in malignant cells during cancer progression. Pluripotent ESCs express high levels of EpCAM that are specifically suppressed during the differentiation to mesodermal lineages, whereas endodermal cells and epithelia retain EpCAM expression (Gonzalez et al., 2009; Lu et al., 2010; Ng et al., 2009; Sarrach et al., 2018). Earliest stages of mesodermal commitment are accompanied by a complete loss of human EpCAM to generate EpCAM^−^/CD56^+^ mesodermal progenitors with the potency to differentiate into hematopoietic, endothelial, mesenchymal, muscle and cardiomyocyte cells (Evseenko et al., 2010). In mice, EpCAM expression is repressed at the initiation of gastrulation in mesodermal progenitors starting at day E7.0 (Sarrach et al., 2018), which is accompanied by a gain of mesodermal markers such as vimentin in human and mouse (Evseenko et al., 2010; Sarrach et al., 2018). Primary carcinomas, metastases, and cancer stem cells express high levels of EpCAM too (Baeuerle and Gires, 2007; Gires et al., 2009; Went et al., 2004), whereas circulating and disseminated tumor cells (CTCs/DTCs) display heterogeneous EpCAM expression with frequent loss during EMT (Brown et al., 2021; Gires and Stoecklein, 2014; Gorges et al., 2012; Keller et al., 2019; Liu et al., 2019). Single-cell analyses of carcinomas of the oral cavity have disclosed a high level of molecular heterogeneity and have identified a subset of cells of primary tumors in a state of partial EMT with a gradual loss of epithelial differentiation (Puram et al., 2017, 2018). EpCAM expression was identified as the major characteristic of retained epithelial differentiation of carcinoma cells (Puram et al., 2017), and loss of EpCAM expression at the edges of tumor areas was frequently accompanied by expression of the mesenchymal marker vimentin (Baumeister et al., 2018). Hence, partial EMT is a central feature of tumor progression that is controlled by tumor-intrinsic programs and cues from the tumor microenvironment (Aggarwal et al., 2021; Ciriello and Magnani, 2021).

**Figure 6:**
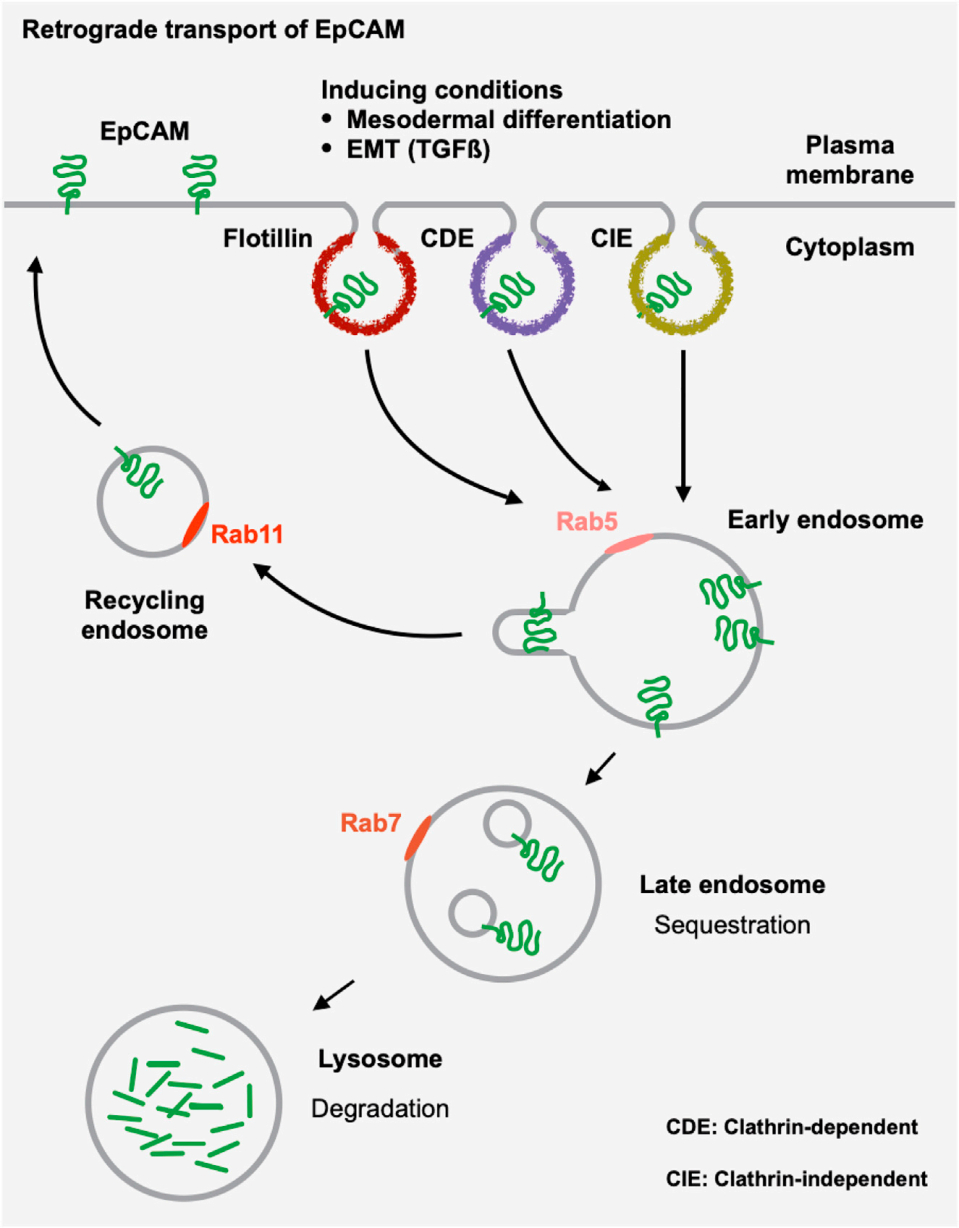
Schematic representation of EpCAM endocytosis and membrane recycling EpCAM endocytosis in early and late endosomes and lysosomes is depicted with the involved Rab proteins. Alternative to degradation in lysosomes, membrane recycling of EpCAM is shown including Rab11.

However, most studies concentrated on the expression of EpCAM at the transcriptional level via epigenetic changes (Lu et al., 2010; Sarrach et al., 2018). The present data shed light on a network of proteins involved in post-translational regulation of EpCAM and its availability at the plasma membrane. Numerous potential EpCAM interactors identified in the present study are instrumental in vesicle-mediated membrane trafficking, including several members of the GTPase family of Rab proteins that are active throughout anterograde and retrograde trafficking of membrane proteins (Figure 6), and are essential in the regulation of cell polarity and migration. Recently, Gaston et al. have shown a central role for EpCAM in remodeling of membranous areas exhibiting enhanced RhoA activity in migrating epithelial cells. Cell polarization, stress fiber formation, and myosin-II activity depend on an EpCAM-mediated spatial distribution of RhoA at the single-cell level through common endosomal trafficking and recycling (Gaston et al., 2021). Our findings further support this reported interaction of EpCAM with RhoA.

EpCAM interactor Rab5c is instrumental in Wnt11-dependent regulation of E-cadherin endocytosis in zebrafish gastrulation to influence mesoendoderm cohesion (Ulrich et al., 2005). Proper development of epithelia (derived from endoderm) in zebrafish depends on EpCAM through an interaction with E-cadherin and the formation of *adherens* junctions (Slanchev et al., 2009). Furthermore, Rab5c is involved in recycling of integrin beta-1, a potential interactor of EpCAM, which is important for invasiveness of breast cancer cells (Onodera et al., 2012). Regulation of the connection of EpCAM, integrin beta-1, and E-cadherin to Rab5c and additional Rab molecules could represent a means of cooperatively controlling the turnover of these proteins to regulate cell adhesion, segregation, and motility in ESC differentiation and metastasis formation.

Accordingly, reduction of hEpCAM was observed in carcinoma cells that adopted a migratory and invasive phenotype (Driemel et al., 2014; Tsaktanis et al., 2015). Despite a distinctive expression pattern of EpCAM during EMT in normal differentiation with an exclusion in mesodermal cells, the actual role of EpCAM in EMT in cancer remains controversially discussed. Both, activating and inhibitory functions of EpCAM in EMT have been described (Gao et al., 2015; Pan et al., 2018; Sankpal et al., 2009, 2011, 2017). Here, we demonstrate a partial loss of EpCAM expression upon mesodermal differentiation of ESC and following induction of EMT by TGFβ in esophageal carcinoma cells. These changes were accompanied by increased endocytosis and reduced recycling of EpCAM. Recently, Wu et al. reported on a matriptase-dependent di-basic cleavage of EpCAM that destabilizes its interaction with the tight junction protein Claudin-7, resulting in endocytosis and lysosomal degradation of EpCAM (Wu et al., 2017). EpCAM interactor Rab14 might additionally be involved in EpCAM turnover through targeting of the ADAM10 protease to the plasma membrane (Linford et al., 2012). ADAM10 is a reported interaction partner of EpCAM (Le Naour et al., 2006) that contributes to the initial cleavage of EpCAM during regulated intramembrane proteolysis (RIP) (Maetzel et al., 2009). We conclude that endocytosis and membrane recycling of EpCAM are post-translational means for the regulation of its availability and, thereby, its function at the plasma membrane during normal and pathologic differentiation (Figure 6).

Lastly, interactions with a wide variety of mitochondrial and nuclear import/export proteins suggest an alternative localization of EpCAM in mitochondria and the nucleus. In fact, top three ranking potential interactors were mitochondrial proteins (prohibitin 1/2 and ATAD3A), and thirteen out of seventy-seven proteins interacting with EpCAM are located in mitochondrial membranes. Although experimental proof is currently lacking, it is tempting to speculate that EpCAM might play a role at the interface of mitochondria and the ER, as was reported for the amyloid precursor protein (APP). APP is present in mitochondria-associated membranes (MAMs), where it becomes cleaved by BACE-1 and the gamma-secretase complex (Del Prete et al., 2017). A connection of EpCAM with the endoplasmic reticulum (ER) in form of an interaction with the ER aminopeptidase 2 (ERAP2) has been reported (Gadalla et al., 2013), although the actual function of this interaction remained unexplored. Proteins involved in nuclear import and export of cargos such as TNPO1 and CSE1L may hint toward a possible nuclear translocation of EpCAM. Co-precipitation of EpCAM with TNPO1 and CSE1L was confirmed in mF9 cells, and EpCAM could be detected in nuclear extracts and by confocal imaging of mF9 and Kyse30 cells (data not shown). However, based on technical drawbacks regarding the contamination of nuclear fraction with membranous components and a current lack of function, final conclusions on a nuclear localization of EpCAM are not feasible.

In summary, the present results provide an insight into the regulation of EpCAM expression during mesodermal differentiation and EMT. Additionally, the findings are a valuable platform for future studies on alternative localizations and functions of EpCAM, which might explain its numerous roles in various cell types.

### Limitations of the study

Limitations of the present study must be considered since combination of immunoprecipitation and SILAC approach failed to enrich α- and β-sheddases, components of the γ-secretase complex, claudins, and intracellular ligands reported for human EpCAM such as FHL2 and β-catenin. Technical limitations related to protein amounts as well as a possibly transient interaction of EpCAM with these proteins may account for the lack of enrichment. Finally, the limited number of different cellular states that can be analyzed via SILAC and issues regarding data analysis based on a potential proline-to-arginine conversion in cells may further impact on how comprehensive the analysis of EpCAM interactors eventually is.

## STAR★Methods

### Key resources table

**Table:** 

REAGENT or RESOURCE	SOURCE	IDENTIFIER
**Antibodies**		

GFP-Trap® agarose beads	ChromoTek, Planegg-Martinsried, Germany	ChromoTek Cat# gta-20, RRID:AB_2631357
Anti-GFP	Santa Cruz, USA	Santa Cruz Biotechnology Cat# sc-9996, RRID:AB_627695
Anti-Prohibitin 1	Santa Cruz, USA	Santa Cruz Biotechnology Cat# sc-28259, RRID:AB_2164486
Anti-Prohibitin 2	Santa Cruz, USA	Santa Cruz Biotechnology Cat# sc-67045, RRID:AB_2283865
Anti-Calnexin	Enzo, USA	Enzo Life Sciences Cat# ADI-SPA-860, RRID:AB_10616095
Anti-Rab5	Cell Signaling, USA	Cell Signaling Technology Cat# 46449, RRID:AB_2799303
Anti-Rab7	Cell Signaling, USA	Cell Signaling Technology Cat# 9367, RRID:AB_1904103
Anti-Rab11	Cell Signaling, USA	Cell Signaling Technology Cat# 5589, RRID:AB_10693925
Anti-murine EpCAM-Alexa-488	Abcam, Cambridge UK	Cat# ab237384
Anti-human EpCAM-Alexa-488	Abcam, Cambridge UK	Cat# ab237395
Anti-Alexa-488	Thermo Fisher Scientific, USA	Thermo Fisher Scientific Cat# A-11094, RRID:AB_221544

**Chemicals, peptides, and recombinant proteins**		

Lysotracker	Thermo Fisher Scientific, USA	Cat# L7528
Mouse ES cell basal medium	ATCC, LGC Standards GmbH, Germany	Cat# SCRR-2011
Embryonic stem cell grade FBS	Bio&SELL GmbH, Germany	Cat# FBS-E

**Critical commercial assays**		

PowerUp SYBR Green Master Mix	Applied Biosystems, USA	Cat# A25742

**Experimental models: cell lines**		

Murine F9	ATCC, USA (Prof. Marcus Conrad)	ATCC Cat# CRL-1720, RRID:CVCL_0259
E14TG2a	ATCC, USA	ATCC Cat# CRL-1821, RRID:CVCL_9108
Kyse30	DSMZ, Germany	DSMZ Cat# ACC-351, RRID:CVCL_1351

**Oligonucleotides**		

Murine GUSB qPCR primers	Metabion, Germany	Forward: CAACCTCTGGTGGCCTTACC Reverse: GGGTGTAGTAGTCAGTCACAGAC
Murine Sox2 qPCR primers	Metabion, Germany	Forward: GACAGCTACGCGCACATGA Reverse: GGTGCATCGGTTGCATCTG
Murine Oct3/4 qPCR primers	Metabion, Germany	Forward: CGGAAGAGAAAGCGAACTAGC Reverse: ATTGGCGATGTGAGTGATCTG
Murine	Metabion, Germany	Forward: TCTTCCTGGTCCCCACAGTTT Reverse: GCAAGAATAGTTCTCGGGATCAA
Murine α-CAA qPCR primers	Metabion, Germany	Forward: CTGGATTCTGGCGATGGTGTA Reverse: CGGACAATTTCACGTTCAGCA
Murine Vimentin qPCR primer	Metabion, Germany	Forward: ACCGGAGCTATCTGACCACG Reverse: CAAGGATTCCAGTTTCCGTTCA
Human GAPDH	Metabion, Germany	Forward: AGGTCGGAGTCAACGGATTT Reverse: TAGTTGAGGTCAATGAAGGG
Human ZEB1	Metabion, Germany	Forward: TTACACCTTTGCATACAGAACCC Reverse: TTTACGATTACACCCAGACTGC
Human ZEB2	Metabion, Germany	Forward: GGAGACGAGTCCAGCTAGTGT Reverse: CCACTCCACCACCCTCCCTTATTTC
Human SNAI1	Metabion, Germany	Forward: AGATGAGCATTGGCAGCGAG Reverse: TGGGAAGCCTAACTACAGCGA
Human SNAI2	Metabion, Germany	Forward: CGAACTGGACACACATACAGTG Reverse: CTGAGGATCTCTGGTTGTGGT
Human TWIST1	Metabion, Germany	Forward: GCTTGAGGGTCTGAATCTTGCT Reverse: GTCCGCAGTCTTACGAGGAG

**Recombinant DNA**		

pCAG-3SIP vector	pCAG vector modified to include 3x Stop in all three reading frames, encephalomyocarditis IRES, and globin polyA tail. Kind gift from Prof. Marcus Conrad (HMGU, Munich, Germany)	https://edoc.ub.uni-muenchen.de/9432/1/Seiler_Alexander.pdf
pCAG-EpCAM-YFP	Cloned by insertion of PCR amplified EpCAM-YFP into EcoR1 cut pCAG-3SIP vector	N/A
pCAG-Ep-CTF-YFP	Cloned by insertion of PCR amplified Ep-CTF-YFP into EcoR1 cut pCAG-3SIP vector	N/A
pCAG-EpICD-YFP	Cloned by insertion of PCR amplified EpICD-YFP into EcoR1 cut pCAG-3SIP vector	N/A
pCAG-YFP	Cloned by insertion of PCR amplified YFP into EcoR1 cut pCAG-3SIP vector	N/A
mCherry-Rab5a	Addgene	Cat# 27679
mCherry-Rab7	Addgene	Cat# 55127
mCherry-Rab11	Addgene	Cat# 55124

**Software and algorithms**		

GraphPad Prism 8	GraphPad Software Inc., USA	N/A
MaxQuant software	https://www.maxquant.org/	http://coxdocs.org/doku.php?id=maxquant:start
CytExpert	Beckman Coulter Diagnostics, USA	

**Other**		

Leukemia inducing factor ESGRO LIF	Merk, Darmstadt, Germany	Cat# ESG1107

### Resource availability

#### Lead contact

Further information and requests for resources and reagents should be directed to and will be fulfilled by the lead contact, Olivier Gires (Olivier.gires@med.uni-muenchen.de).

#### Materials availability

This study did not generate new unique reagents.

### Experimental model and subject details

#### Cell lines

Murine F9 (mF9; male) cells were cultured in Dulbecco's modified Eagle medium (DMEM, high glucose) in the presence of 20% FCS (Biochrom AG, Heidelberg, Germany). Pluripotency of E14TG2a cells (male) was achieved through culture on gelatin-treated culture plates (0.1%, InScreeenEx, Braunschweig, Germany) in Mouse ES Cell Basal Medium (ATCC, SCRR-2011, LGC Standards GmbH, Germany) supplemented with 1,000U/mL leukemia inducing factor ESGRO® LIF (Merck, Darmstadt, Germany), 10% embryonic stem cell grade FBS (FBS-E, Bio&SELL GmbH, Germany) and 0.1nM 2-mercaptoethanol (Thermo Fischer Scientific, MA, USA). For all experiments including E14TG2a ESC, cells in passages below 60, with a colony-forming morphology, and high expression of pluripotency markers Oct3/4, Sox, and Nanog were used. Kyse30 carcinoma cells (male) were obtained from DSMZ and were confirmed by STR typing using ten markers (AMEL, CSF1PO, D13S317, D16S539, D21S11, D5S818, D7S820, TH01, TPOX, vWA). Kyse30 cells were maintained in RPMI 1640, 10% FCS, 1% penicillin/streptomycin, in a 5% CO2 atmosphere at 37°C.

### Method details

#### Transfections, vectors, flow cytometry

All expression plasmids are based on the 141pCAG-3SIP vector. Using EcoR1 and Nhe1 sites the following coding sequences were inserted following PCR amplification: yellow fluorescence protein (YFP), full-length murine EpCAM (314 aa) fused to YFP (C-terminal), murine EpCAM C-terminal fragment consisting of the signal peptide of EpCAM (aa 1–23) a short linker (lysin, leucin), a Myc tag, and the C-terminal fragment (aa 251–314) fused to YFP (C-terminal), and the EpCAM intracellular domain (26 aa) fused to YFP (Hachmeister et al., 2013). Transfection of expression vectors was conducted with MATra reagent (Iba, Goettingen, Germany) (mF9) or the Amaxa nucleofector kit (Lonza, Ratingen, Germany) (E14TG2α cells). Stable expression was achieved through selection with puromycin (4μg/mL). Bafilomycin A1 (10 nM; Cell Signaling Technology, Frankfurt, Germany) and DAPT (10μM; Sigma Aldrich, Taufkirchen, Germany) treatment of cells was done for a time of 10-24hrs. Fluorescence of YFP and fusion proteins was assessed in a FACScalibur device in the FLH1 channel (Becton Dickinson, Heidelberg, Germany).

#### Co-immunoprecipitation and immunoblot

For co-immunoprecipitation, 4mg of total protein lysate were subjected to GFP-mediated immunoprecipitation of YFP and fusion proteins using GFP-Trap® agarose beads (ChromoTek, Planegg-Martinsried, Germany). Superior expression of YFP compared to EpCAM-YFP was adjusted through the addition of wild-type mF9 cell lysate to YFP lysates. Precipitated proteins were washed with ice-cold washing buffer containing 0.5% tween ×100 in TBS, resuspended in 20μL Laemmli buffer (Laemmli, 1970), separated in a 10%-SDS-PAGE, transferred onto activated PVDF membrane (Millipore, Darmstadt, Germany) and detected with GFP (Santa Cruz, sc-9996; USA), prohibitin-1 (Santa Cruz, sc-28259; USA), prohibitin-2 (Santa Cruz, sc-67045; USA), calnexin (Enzo, ADI-SPA-860; USA), Rab5 (Cell Signaling, E6N8S; USA), Rab7 (Cell Signaling, D95F2; USA) and Rab11 (Cell Signaling, D4F5; USA) specific antibodies in conjunction with HRP-conjugated secondary antibodies and ECL (Millipore, Darmstadt, Germany).

#### Confocal laser scanning microscopy

Fluorescence was analyzed in mF9 cells stably transfected with YFP, EpCAM-YFP, mCTF-YFP, or mEpICD-YFP, and transiently transfected with mCherry-tagged versions of Rab5, Rab7, and Rab11 (Addgene; #27679, #55127, #55124). For live cell imaging, cells were plated on Ibidi 8-well glass bottom μ-slides (Ibidi, Matrinsried, Germany; # 80827), stained with 10 μg/ml Hoechst33342 (Sigma, Germany #94403) and imaged in phenol red-free RPMI1640 (Thermo Fisher Scientific, #11835030). Rab5 (Cell Signaling, E6N8S; USA), Rab7 (Cell Signaling, D95F2; USA), and Rab11 (Cell Signaling, D4F5; USA) were stained with specific antibodies in combination with phycoerythrin-conjugated secondary antibodies. Lysosomes were stained with 19 nM Lysotracker (Thermo Fisher Scientific, L7528; USA) for 30 minutes in the respective culture medium. Fluorescence was visualized immediately using a TCS-SP8 scanning system, a DM-IRB inverted microscope using a 63x oil objective with a NA of 1.4 and the LAS AF software (Leica, Nussloch, Germany).To visualize endocytosed EpCAM, mF9, E14TG2a, and Kyse30 cells were plated as described above and subjected to the internalization assay described below, and samples were taken after staining with an anti-murine of -human EpCAM-Alexa-488 antibody (Abcam, Cambridge, UK; ab237384 and #ab237395), after quenching with an anti-Alexa-488 antibody, and after 30 min of internalization. Alexa-488 fluorescence was imaged using a TCS-SP8 scanning system, a DM-IRB inverted microscope using a 63x oil objective with a NA of 1.4 and LAS AF software (Leica, Nussloch, Germany).

Brightness and contrast of microscopic images were adjusted linearly where indicated in figure legends and scale bars were added using Fiji version 1.52i (Schindelin et al., 2012). Colocalization analysis was performed using the JACoP plugin version 2.1.1 (Bolte and Cordelieres, 2006) with the function “M1 & M2 coefficients” and manually adjusted thresholds.

#### KEGG and GO-term classification

Potential EpCAM interactors analysis was performed using STRING (v.11.0) to scrutiny Kyoto Encyclopedia of Genes and Genomes (KEGG) pathways and Gene Ontology (GO) terms, including biological process (BP), cellular component (CC), and molecular function (MF). Enrichment analysis were applied based on the Fisher’ exact test, considering the whole quantified interaction proteins as background dataset. Benjamini-Hochberg correction for multiple testing was further applied to adjust p-value, and only functional categories and pathways with p value <0.05 were considered significant.

#### Mesodermal differentiation and EMT induction

Guided mesodermal differentiation of pluripotent murine embryonic stem cells was induced as described by Sarrach et al. (Sarrach et al., 2018) *via* a modified protocol from Kanke et al. (Kanke et al., 2014). For this protocol, E14TG2α cells were seeded in 6 well plates (100.000 cells/well) in Mouse ES Cell Basal Medium supplemented with 1000U/mL ESGRO® LIF, 10% stem cell grade FBS and 0.1nM 2-mercaptoethanol 24 hours before start of differentiation. To induce mesodermal differentiation, cells were thoroughly washed with PBS and cultured in differentiation medium w/o LIF (Mouse ES Cell Basal Medium, 10% stem cell grade FBS, 0.1nM 2-mercaptoethanol) with 30μM CHIR99021 (Sigma, St. Louis, MO, USA) and 5 μM Cyclopamine (Selleckchem, Houston, USA) at 37°C and 5% CO_2_. After 5 days incubation, cells were harvested with Trypsin for further processing.

Induction of epithelial-to-mesenchymal transition (EMT) in Kyse30 cells was performed as follows: cells were seeded in 6 well plates (100.000 cells/well) in complete medium for 24 hours, after which the medium was removed, cells washed with PBS, and cultured in FCS-free medium for another 24 hours. EMT was induced by adding recombinant TGFβ (Abcam, Cambridge, UK, #ab50036) at 20 ng/mL and cells were harvested 48 hours later for further processing.

#### Internalization and membrane recycling

The assay was performed *via* a modified protocol from Arjonen et al. (Arjonen et al., 2012). Murine F9 cells, pluripotent and mesodermally differentiated E14TG2a cells, and control- and TGFβ-treated Kyse30 cells were harvested and washed twice with cold cell staining buffer (PBS with 3% stem cell grade FBS). Cells were then incubated with an anti-EpCAM AF488-conjugated antibody (ab237384, Abcam, Cambridge, UK) 1:50 in 400μL cell staining buffer for 1 hour at 4°C in the dark, after which they were washed 3 times with cell staining buffer. Samples were then divided into 4 parts: 100% labeled control, quenching background control, endocytosis group, and recycling group. The 100% labeled control can be directly analyzed by flow cytometry (Beckman Coulter Cytoflex device, Germany). The quenching background group was incubated with an anti-AF488 antibody (A-11094, Thermo Fisher Scientific, MA, USA) 1:50 in 100μL cell staining buffer for 1 hour at 4°C in the dark to quench cell surface fluorescence. After washing 3 times with cell staining buffer, cells were analyzed by flow cytometry. The endocytosis and recycling groups were suspended in 1mL cell culture medium and incubated at 37°C for 30 mins to allow internalization of cell surface EpCAM. Cells were then washed twice with cold cell staining buffer and the cell surface fluorescence was quenched by incubation with the anti-AF488 antibody. For analysis of endocytosis in mF9 cells, samples were assessed every 5 mins. After washing 3 times with cold cell staining buffer, the endocytosis group was analyzed by flow cytometry. The recycling group was again incubated at 37°C for 30 mins to allow recycling of internalized EpCAM to the membrane, after which the cell surface fluorescence was again quenched and the cells analyzed by flow cytometry. Recycling rate was measured every 10 mins for mF9 cells. To calculate endocytosis and recycling rates, live cells were gated and Mean Fluorescence Intensities (MFI) were normalized against unstained controls (MFI(sample)-MFI(unstained control)), after which the quenching background was subtracted from all samples (MFI(sample)-MFI(quenching background)). To calculate endocytosis rates, MFI of the samples were normalized to the 100% labeled control (MFI(endocytosis group)/MFI(100% labeled)), while recycling rates were calculated by subtracting the MFI of the endocytosis group and normalizing to the same ((MFI(recycling)-MFI(endocytosis))/MFI(endocytosis)).

#### Quantitative real-time polymerase chain reaction

Total mRNA was isolated using the RNeasy Mini Kit (QIAGEN, Hilden, Germany) and reverse transcribed with the QuantiTect Reverse Transcription Kit (QIAGEN, Hilden, Germany) according to the manufacturer’s instructions. Quantitative PCR was performed using the PowerUp SYBR Green Master Mix (Applied Biosystems, MA, USA) in a volume of 10μl using gene-specific primers on a QuantStudio™ 3 Real-Time PCR System (Thermo Fisher Scientific, MA, USA). Samples were normalized to the housekeeping gene Glucoronidase Beta (GUSB) and relative gene expression was calculated using the delta delta Ct (ΔΔCt) formula. Real time primer sequences are shown below.

**Table:** 

Gene	Forward primer 5’→ 3’	Reverse primer 3’→ 5’
*GUSB*	CAACCTCTGGTGGCCTTACC	GGGTGTAGTAGTCAGTCACAGAC
*Sox2*	GACAGCTACGCGCACATGA	GGTGCATCGGTTGCATCTG
*Oct3/4*	CGGAAGAGAAAGCGAACTAGC	ATTGGCGATGTGAGTGATCTG
*Nanog*	TCTTCCTGGTCCCCACAGTTT	GCAAGAATAGTTCTCGGGATCAA
*α-CAA*	CTGGATTCTGGCGATGGTGTA	CGGACAATTTCACGTTCAGCA
*Vimentin*	ACCGGAGCTATCTGACCACG	CAAGGATTCCAGTTTCCGTTCA

### Quantification and statistical analysis

#### SILAC analysis

The general procedure of the SILAC screen implementing mF9 cells expressing EpCAM-YFP or YFP has been described elsewhere (Sarrach et al., 2018). Briefly, mF9 cells stably expressing EpCAM-YFP or YFP proteins were cultured 14 days (representing 3–5 passages) in medium (Silantes, Munich, Germany) containing heavy (lysine-8/arginine-10) and light amino acids (lysine-0/arginine-0), respectively. In three biological repeats, 3mg (exp. #1 and #2) or 7mg (exp.#3) whole cell lysate were incubated with 30μL GFP-Trap® agarose beads (3 hrs, 4°C, rotation), washed in 700μl 0.2% tween in PBS. Independent samples (n = 3) from EpCAM-YFP and YFP immunoprecipitants were pooled and proteins recovered upon heating (95°C, 5 min) in Laemmli buffer (Laemmli, 1970). Immunoprecipitated proteins were separated on SDS-PAGE, trypsinized by in-gel digestion, and analyzed *via* LC-MS/MS on a LTQ Orbitrap XL coupled to an Ultimate 3000 nano-HPLC. SILAC data analysis was performed using the MaxQuant software (Merl et al., 2012). Potential interaction partners were defined as proteins enriched by ≥ 3-fold with p values ≤0.05 and ≥2 unique peptides in all independent experiments. Two-sided unpaired t-tests were conducted on individual protein intensities for each label and sample (intensities for replicate #3 were adjusted for differences in protein input in the IP). All proteomic data are compiled in supplementary Excel file Data S1.

Statistical analysis was performed in GraphPad Prism 8 and is indicated in figures and figure legends including numbers of independent experiments (n), statistical tests used, and the level of significance. Data are presented as mean with SD where indicated.

## Data Availability

Data Raw, processed, and analyzed data from SILAC experiments are provided as a supplementary Excel file termed Data S1. All results are summarized to include protein accession numbers, numbers of peptides, oxidation, MWs, ratios and normalized ratios of heavy and light amino acid-marked peptides, intensities, significance values (normalized t-test), protein functions and clustering. Code This paper does not report original code. Any additional information required to reanalyze the data reported in this paper is available from the lead contact upon request.

## Supplemental information

Document S1. Figures S1–S3 and Tables S1–S3

Data S1. Raw data, analyses, SILAC ratios, cellular localization, functional clustering, and proteins groups resulting from the SILAC screen are listed, related to Table 1
