# Supplementary material for: Interactome analysis reveals endocytosis and membrane recycling of EpCAM during differentiation of embryonic stem cells and carcinoma cells
Source: iScience. 2021 Sep 27;24(10):103179. doi: 10.1016/j.isci.2021.103179 (PMC8517208; doi:10.1016/j.isci.2021.103179)
Supplement: Document S1. Figures S1–S3 and Tables S1–S3 [file mmc1.pdf]

iScience, Volume *24*

Supplemental information

Interactome analysis reveals endocytosis and membrane recycling of EpCAM during differentiation of embryonic stem cells and carcinoma cells

Min Pan, Vera Kohlbauer, Alexandra Blancke Soares, Henrik Schinke, Yuanchi Huang, Gisela Kranz, Tanja Quadt, Matthias Hachmeister, and Olivier Gires

Content:

- **Supplementary Figure 1 (related to Figure 3):** Endocytosis of the C-terminal fragment of EpCAM
- **Supplementary Figure 2 (related to Figure 4):** EpCAM and Rab 5, Rab7, Rab11 co-staining
- **Supplementary Figure 3 (related to Figures 4 and 5):** Confocal laser scanning microscopy visualization of EpCAM endocytosis
- **Supplementary Table 1 (related to Table 1):** Gene ontology (GO) terms ”Biological Processes” of putative mEpCAM-YFP interacting proteins.
- **Supplementary Table 2 (related to Table 1):** Gene ontology (GO) terms ”Cellular components” of putative mEpCAM-YFP interacting proteins.
- **Supplementary Table 3 (related to Table 1):** Gene ontology (GO) terms ”Molecular functions” of putative mEpCAM-YFP interacting proteins.

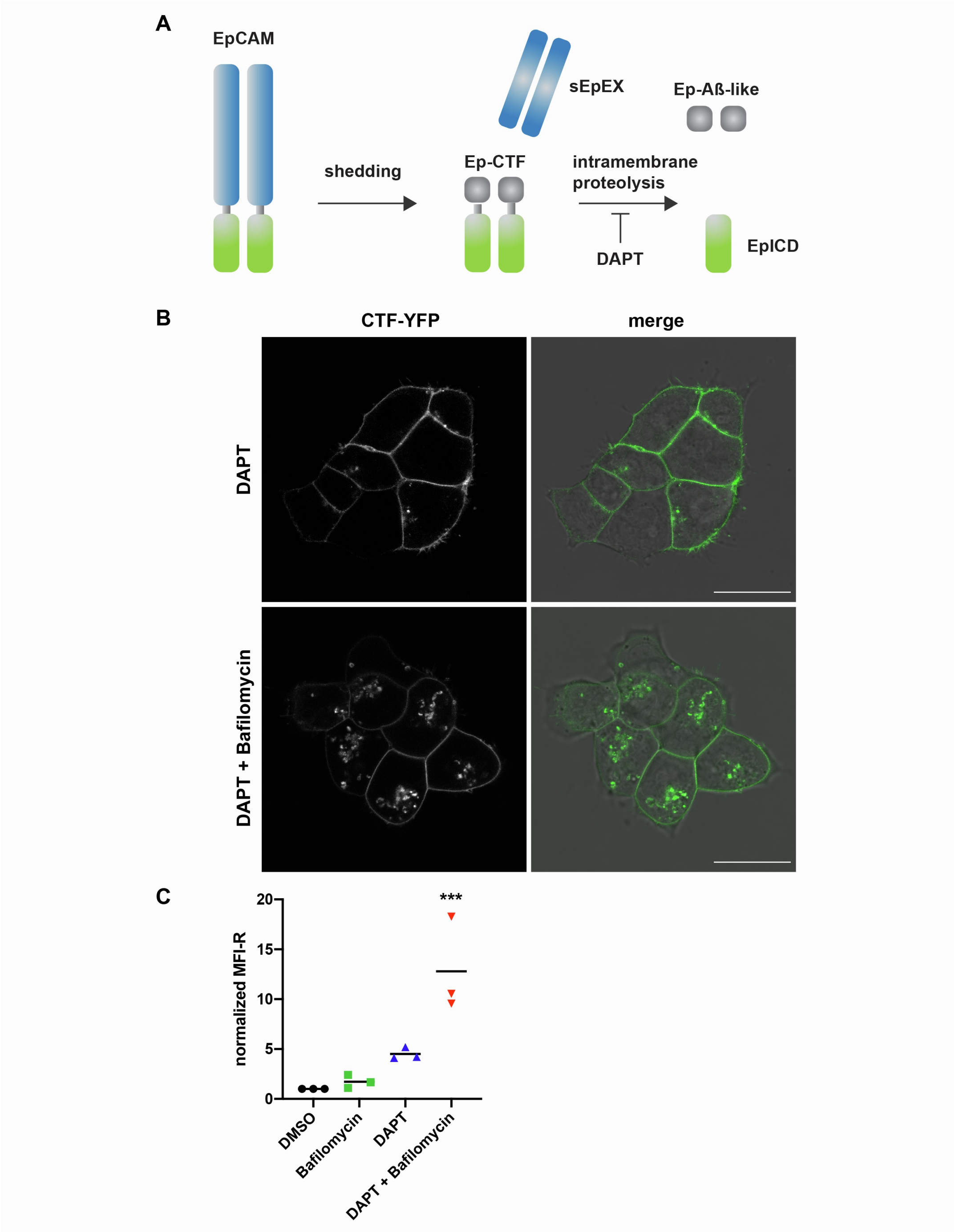

Supplementary Figure 1 (related to Figure 3): Endocytosis of the C-terminal fragment of EpCAM

(**A**) Schematic representation of EpCAM regulated intramembrane proteolysis. EpCAM is subject to shedding of the extracellular domain EpEX to generate the C-terminal fragment Ep-CTF, which is further cleaved through intramembrane proteolysis resulting in the formation of the intracellular domain EpICD and the extracellular Ep-Ab-like fragment. (**B**) mF9 cells stably transfected with Ep-CTF-YFP were treated with DMSO or bafilomycin A1 (10 nM) in combinations with the gamma-secretase inhibitor DAPT (10µM). YFP fluorescence was visualized by confocal laser scanning microscopy. Shown are representative pictures with magnifications. Size scale represents 20 µm. (**C**) mF9 cells stably transfected with Ep-CTF-YFP were treated with DMSO, bafilomycin A1 (10 nM), DAPT (10 µM), or a combination of DAPT and bafilomycin A1. Mean fluorescence intensity ratios were assessed by flow cytometry. Shown are the results from 3-4 independent experiments. Mean values are indicated by a line. One-way ANOVA with DMSO as reference, p-value: *** ≤ 0.001.

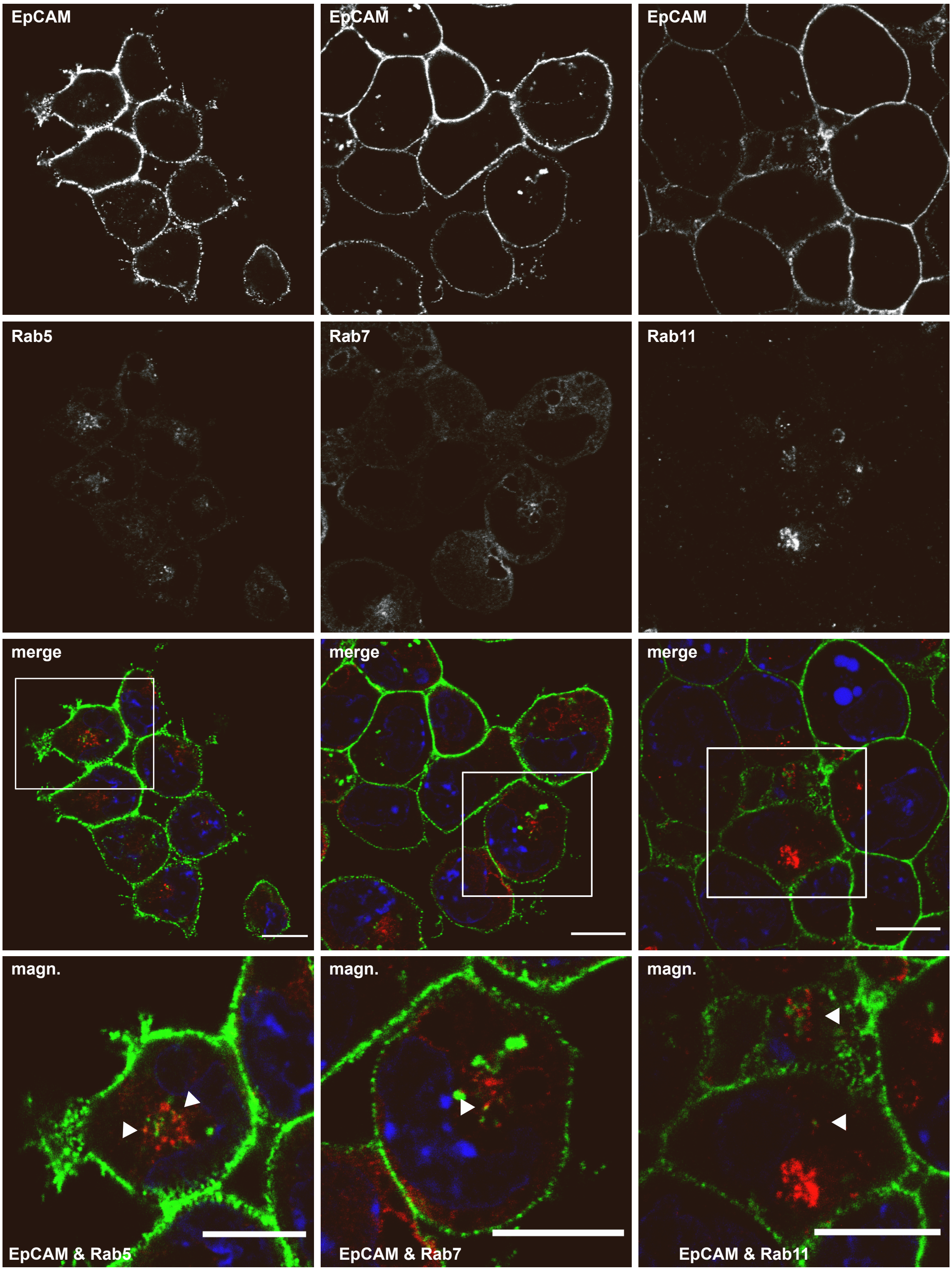

Supplementary Figure 2 (related to Figure 4): EpCAM and Rab 5, Rab7, Rab11 co-staining

mF9 cells were treated with Bafilomycin A1 (10 nM) and stained with EpCAM-, Rab5-, Rab7-, and Rab11-specific antibodies as indicated in combination with Alexa-488- (EpCAM) and Alexa-594-conjugated secondary antibodies (Rab proteins). Fluorescence was visualized by confocal laser scanning microscopy. Shown are representative pictures with magnifications (magn.). Size scale represents 10 µm in the overview and 20 µm in magnifications.

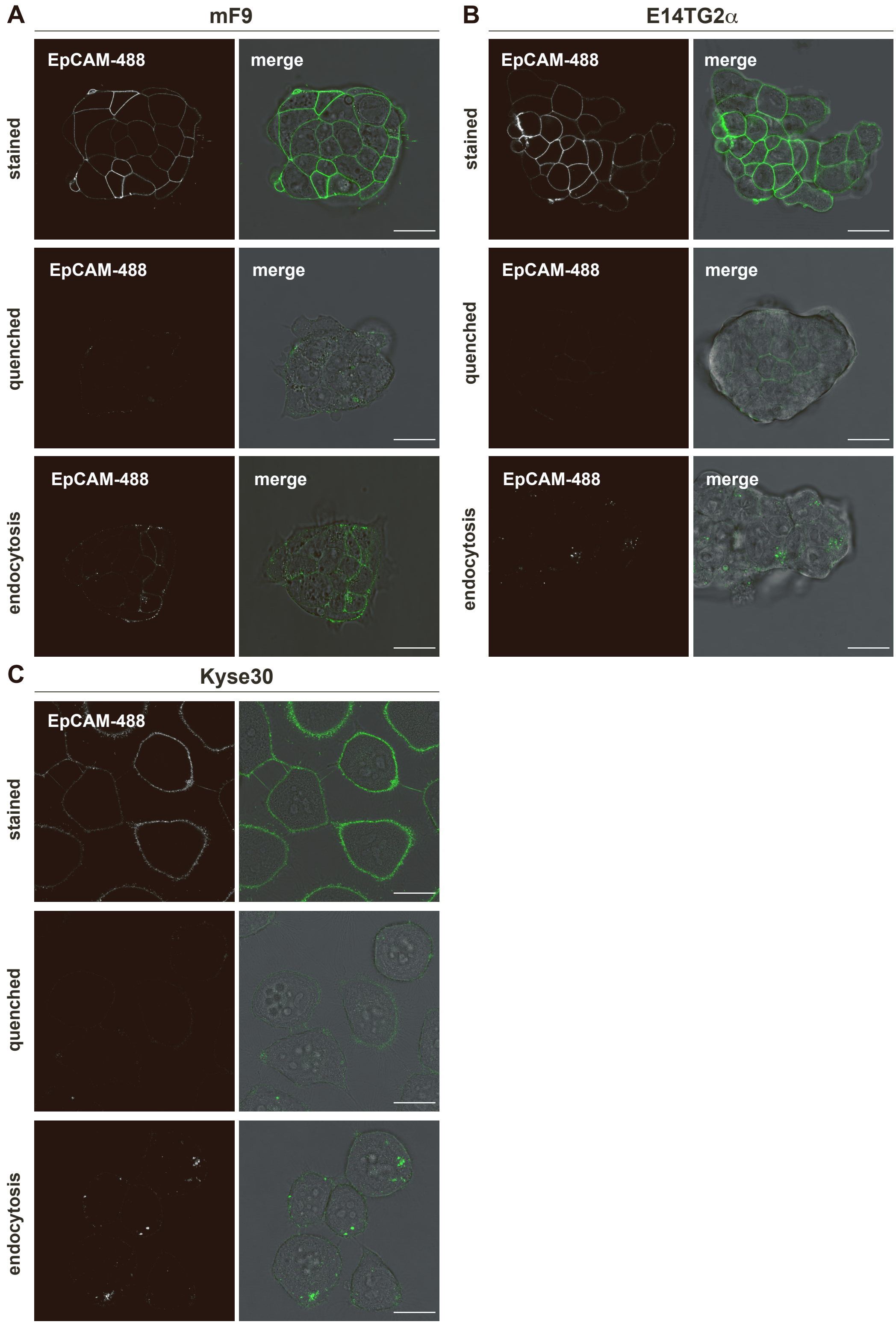

Supplementary Figure 3 (related to Figure 4 and 5): Confocal laser scanning microscopy visualization of EpCAM endocytosis

(**A-B**) Wild-type mF9 (**A**) and E14TG2α cells (**B**) were stained with Alexa-488-labelled anti-EpCAM antibody (stained). Efficiency of fluorescence quenching with an anti-Alexa-488 antibody is shown in the middle panels (quenched). Following staining with anti-EpCAM-Alex-488 antibody, endocytosis was allowed for 30 min (see STAR Methods), cells were washed, and residual Alexa-488 fluorescence at the plasma membrane was quenched with an anti-Alexa-488 antibody (endocytosis). Alexa-488 fluorescence was visualized by confocal laser scanning microscopy for each indicated condition. Shown are representative pictures.

Supplementary Table 1 (related to Table 1): Gene ontology (GO) terms "Biological Processes" of putative mEpCAM-YFP interacting proteins. Shown are the 30 GO terms with highest enrichment levels.

GO term	GO term ID	p-value	Adj. p-value	#Genes observed	#Genes expected	#Genes total
Establishment of localization	GO:0051234	3.87E-17	0.00E+00	47	13.8	4,232
Transport	GO:0006810	6.85E-17	0.00E+00	46	13.3	4,087
Localization	GO:0051179	1.02E-14	0.00E+00	49	17.3	5,316
Vesicle-mediated transport	GO:0016192	3.88E-13	0.00E+00	23	3.6	1,098
Small GTPase-mediated signal transduction	GO:0007264	2.18E-12	0.00E+00	16	1.5	471
Organic substance transport	GO:0071702	1.24E-11	0.00E+00	30	7.6	2,341
Single-organism localization	GO:1902578	2.75E-11	0.00E+00	34	10.2	3,130
Establishment of protein localization	GO:1902578	3.52E-11	0.00E+00	26	5.8	1,798
Protein localization	GO:0045184	3,75E-11	0.00E+00	29	7.4	2,278
Cellular localization	GO:0008104	3.84E-11	0.00E+00	28	6.9	2,117
Macromolecule localization	GO:0051641	7.11E-10	0.00E+00	29	8.4	2,576
Single-organism transport	GO:0033036	7,39E-10	0.00E+00	31	9.6	2,946
Endosomal transport	GO:0044765	1,71E-09	0.00E+00	10	0.7	211
Endomembrane system organization	GO:0016197	4,62E-09	0.00E+00	13	1.5	478
Vacuole organization	GO:0010256	5,77E-09	0.00E+00	9	0.5	175
Vacuolar transport	GO:0007033	6,16E-09	0.00E+00	10	0.8	241
Protein transport	GO:0007034	6,96E-09	0.00E+00	22	5.35	1,639
Single-organism process	GO:0015031	1,43E-08	0.00E+00	66	43	13,200
Cellular protein localization	GO:0044699	1,65E-08	0.00E+00	20	4.6	1,412
Cellular macromolecule localization	GO:0034613	1,81E-08	0.00E+00	20	4.6	1,420
Intracellular signal transduction	GO:0070727	2,03E-08	0.00E+00	25	7.3	2,241
Single-organism cellular localization	GO:0035556	3,18E-08	0.00E+00	16	2.9	911
Endocytosis	GO:1902580	8,22E-08	0.00E+00	12	1.6	504
Establishment of localization in cell	GO:0006897	1,36E-07	0.00E+00	20	5.2	1,605
Regulation of vesicle-mediated transport	GO:0051649	1,57E-07	0.00E+00	11	1.4	433
Intracellular transport	GO:0060627	2,33E-07	0.00E+00	17	3.9	1,196
Exocytosis	GO:0006887	4,78E-07	0.00E+00	9	0.9	293
Receptor metabolic process	GÖ:0043112	7,21E-07	0.00E+00	7	0.5	154
Cell communication	GO:0007154	1,64E-06	0.00E+00	39	19.5	5,997
Golgi vesicle transport	GO:0048193	1,83E-06	0.00E+00	7	0.6	177

Supplementary Table 2 (related to Table 1): Gene ontology (GO) terms "Cellular components" of putative mEpCAM-YFP interacting proteins. Shown are the 30 GO terms with highest enrichment levels.

GO term	GO term ID	p-value	Adj. p-value	#Genes observed	#Genes expected	#Genes total
Membrane	GO:0016020	5.81E-22	0.00E+00	70	30	9205
Extracellular exosome	GO:0070062	6,17E-21	0.00E+00	42	8,5	2590
Extracellular vesicle	GO:1903561	7,47E-21	0.00E+00	42	8,5	2603
Extracellular organelle	GO:0043230	8,28E-21	0.00E+00	42	8,5	2610
Membrane-bounded vesicle	GO:0031988	1,99E-20	0.00E+00	45	10,3	3172
Endomembrane system	GO:0012505	3,71E-20	0.00E+00	46	11,1	3400
Cytoplasmic part	GO:0044444	7,19E-20	0.00E+00	60	21,5	6606
Vesicle	GO:0031982	9,22E-20	0.00E+00	46	11,3	3476
Organelle membrane	GO:0031090	9,18E-19	0.00E+00	32	4,9	1499
Whole membrane	GO:0098805	2,23E-18	0.00E+00	27	3,2	985
Bounding membrane of organelle	GO:0098588	3,40E-18	0.00E+00	26	2,9	903
Extracellular region part	GO:0044421	7,54E-17	0.00E+00	44	12,1	3704
Cytoplasm	GO:0005737	1,84E-16	0.00E+00	67	32,5	9966
Intracellular organelle part	GO:0044446	2,10E-15	0.00E+00	53	19,8	6084
Organelle part	GO:0044422	7,56E-15	0.00E+00	53	20,4	6259
Extracellular region	GO:0005576	1,44E-14	0.00E+00	44	13,9	4255
Cell part	GO:0044464	1,15E-13	0.00E+00	75	48,9	14997
Cell	GO:0005623	1,19E-13	0.00E+00	75	48,9	15005
Intracellular membrane-bounded organelle	GO:0043231	2,17E-13	0.00E+00	64	32,95	10103
Membrane-bounded organelle	GO:0043227	3,14E-13	0.00E+00	67	36,8	11285
Cytoplasmic, membrane-bounded vesicle	GO:0016023	4,19E-13	0.00E+00	20	2,5	776
Endosome	GO:0005768	7,81E-13	0.00E+00	19	2,3	703
Intracellular organelle	GO:0043229	7,85E-13	0.00E+00	66	36,1	11076
Vacuolar membrane	GO:0005774	2,42E-12	0.00E+00	14	1,05	324
Organelle	GO:0043226	3,39E-12	0.00E+00	68	39,6	12156
Vacuolar part	GO:0044437	4,45E-12	0.00E+00	14	1,1	339
Vacuole	GO:0005773	7,57E-12	0.00E+00	21	3,34	1024
Endocytic vesicle	GO:0030139	7,61E-12	0.00E+00	10	0,4	122
Intracellular part	GO:0044424	1,09E-11	0.00E+00	69	41,8	12819
intracellular	GO:0005622	1,83E-11	0.00E+00	69	42,2	12929

Supplementary Table 3 (related to Table 1): Gene ontology (GO) terms "Molecular functions" of putative mEpCAM-YFP interacting proteins. Shown are the 30 GO terms with highest enrichment levels.

GO term	GO term ID	p-value	Adj. p-value	#Genes observed	#Genes expected	#Genes total
GDP binding	GO:0019003	4,10E-19	0.00E+00	12	0.18	58
GTPase activity	GO:0003924	2,64E-14	0.00E+00	13	0,59	184
GTP binding	GO:0005525	2,65E-13	0.00E+00	15	1,10	343
Guanyl ribonucleotide binding	GO:0032561	6,24E-13	0.00E+00	15	1,17	364
Guanyl nucleotide binding	GO:0019001	6,49E-13	0.00E+00	15	1,18	365
Protein binding	GO:0005515	3,92E-10	0.00E+00	52	25,57	7903
Nucleoside-triphosphate activity	GO:0017111	4,93E-10	0.00E+00	16	2,22	687
Myosin binding	GO:0017022	9,36E-10	0.00E+00	7	0,19	60
Pyrophosphate activity	GO:0016462	1,12E-09	0.00E+00	16	2,35	727
Hydrolase activity, acting on anhydrides, In phosphorus-containing anhydrides	GO:0016818	1,16E-09	0.00E+00	16	2,35	729
Hydrolase activity, acting on anhydrides	GO:0016817	1,18E-09	0.00E+00	16	2,36	730
Enzyme binding	GO:0019899	2,00E-09	0.00E+00	24	6,02	1862
Carbohydrate derivate binding	GO:0097367	3,36E-09	0.00E+00	25	6,72	2077
Myosin V binding	GO:0031489	3,47E-09	0.00E+00	5	0,06	19
Ribonucleotide binding	GO:0032553	8,28E-08	0.00E+00	21	5,62	1737
Heterocyclic compound binding	GO:1901363	2,08E-07	0.00E+00	37	16,68	5155
Purine ribonucleoside triphosphate binding	GO:0035639	2,22E-07	0.00E+00	20	5,41	1674
Purine ribonucleoside binding	GO:0032550	2,54E-07	0.00E+00	20	5,46	1688
Ribonucleoside binding	GO:0032549	2,59E-07	0.00E+00	20	5,46	1690
Purine nucleoside binding	GO:0001883	2,61E-07	0.00E+00	20	5,47	1691
Nucleoside binding	GO:0001882	2,79E-07	0.00E+00	20	5,49	1698
Binding	GO:0005488	3,14E-07	0.00E+00	61	40,12	12400
Organic cyclic compound binding	GO:0097159	3,25E-07	0.00E+00	37	16,96	5242
Purine ribonucleotide binding	GO:0032555	3,50E-07	0.00E+00	20	5,57	1722
Purine nucleotide binding	GO:0017076	3,83E-07	0.00E+00	20	5,60	1732
Nucleotide binding	GO:0000166	7,25E-07	0.00E+00	22	6,98	2158
Nucleoside phosphate binding	GO:1901265	7,25E-07	0.00E+00	22	6,98	2158
Small molecule binding	GO:0036094	7,29E-07	0.00E+00	23	7,58	2344
Ran GTPase binding	GO:0008536	3,39E-06	0.00E+00	4	0,10	32

Glycoprotein binding	GO:0001948	4,24E-05	0.00E+00	5	0,38	119
